# A LASSO-based approach to sample sites for phylogenetic tree search

**DOI:** 10.1093/bioinformatics/btac252

**Published:** 2022-06-27

**Authors:** Noa Ecker, Dana Azouri, Ben Bettisworth, Alexandros Stamatakis, Yishay Mansour, Itay Mayrose, Tal Pupko

**Affiliations:** The Shmunis School of Biomedicine and Cancer Research, George S. Wise Faculty of Life Sciences, Tel Aviv University, Tel Aviv 69978, Israel; The Shmunis School of Biomedicine and Cancer Research, George S. Wise Faculty of Life Sciences, Tel Aviv University, Tel Aviv 69978, Israel; School of Plant Sciences and Food Security, George S. Wise Faculty of Life Sciences, Tel Aviv University, Tel Aviv 69978, Israel; Computational Molecular Evolution Group, Heidelberg Institute for Theoretical Studies, 69118 Heidelberg, Germany; Institute of Theoretical Informatics, Karlsruhe Institute of Technology, 76128 Karlsruhe, Germany; Computational Molecular Evolution Group, Heidelberg Institute for Theoretical Studies, 69118 Heidelberg, Germany; Institute of Theoretical Informatics, Karlsruhe Institute of Technology, 76128 Karlsruhe, Germany; The Blavatnik School of Computer Science, Raymond & Beverly Sackler Faculty of Exact Sciences, Tel Aviv University, Tel Aviv 69978, Israel; School of Plant Sciences and Food Security, George S. Wise Faculty of Life Sciences, Tel Aviv University, Tel Aviv 69978, Israel; The Shmunis School of Biomedicine and Cancer Research, George S. Wise Faculty of Life Sciences, Tel Aviv University, Tel Aviv 69978, Israel

## Abstract

**Motivation:**

In recent years, full-genome sequences have become increasingly available and as a result many modern phylogenetic analyses are based on very long sequences, often with over 100 000 sites. Phylogenetic reconstructions of large-scale alignments are challenging for likelihood-based phylogenetic inference programs and usually require using a powerful computer cluster. Current tools for alignment trimming prior to phylogenetic analysis do not promise a significant reduction in the alignment size and are claimed to have a negative effect on the accuracy of the obtained tree.

**Results:**

Here, we propose an artificial-intelligence-based approach, which provides means to select the optimal subset of sites and a formula by which one can compute the log-likelihood of the entire data based on this subset. Our approach is based on training a regularized Lasso-regression model that optimizes the log-likelihood prediction accuracy while putting a constraint on the number of sites used for the approximation. We show that computing the likelihood based on 5% of the sites already provides accurate approximation of the tree likelihood based on the entire data. Furthermore, we show that using this Lasso-based approximation during a tree search decreased running-time substantially while retaining the same tree-search performance.

**Availability and implementation:**

The code was implemented in Python version 3.8 and is available through GitHub (https://github.com/noaeker/lasso_positions_sampling). The datasets used in this paper were retrieved from Zhou *et al*. (2018) as described in section 3.

**Supplementary information:**

Supplementary data are available at *Bioinformatics* online.

## 1 Introduction

Phylogenetic tree inference is a fundamental challenge in evolutionary research. Phylogenetic trees can be statistically inferred from a multiple sequence alignment (MSA), a set of DNA/protein sequences, for which homology was inferred at the single residue resolution. Advanced phylogenetic inference methods are based on the phylogenetic likelihood function, which is the probability of observing the sequence data given a phylogenetic tree under a probabilistic model of sequence evolution. It was previously shown that identifying the tree that maximizes the likelihood is NP-hard (Chor and Tuller, 2005), and thus applying heuristic-search strategies to explore the vast tree space is required to infer hopefully near-optimal trees (Felsenstein, 2004). Most heuristic maximum-likelihood (ML) search strategies rely on a hill-climbing optimization technique. Employing this technique requires an iterative evaluation of possible modifications to the current tree, which are termed neighboring trees. The search can start from a random tree, or more commonly from a tree generated by fast algorithms, e.g. the distance-matrix method or parsimony (Felsenstein, 1981). The search terminates when reaching a local maximum, i.e. when the current tree has no neighbors with a higher likelihood score.

There are several variants of the hill-climbing search. For example, there are several alternative definitions of neighboring trees, including the nearest neighbor interchange (NNI) (Moore *et al.*, 1973; Robinson, 1971), which is used in, for example, IQ-TREE (Nguyen *et al.*, 2015), the lazy subtree pruning and regrafting (SPR) (Felsenstein, 2004), which is used in RAxML-NG (Kozlov *et al.*, 2019) and in PhyML (Guindon *et al.*, 2010) and the tree bisection and regrafting (TBR) (Allen and Steel, 2001), which provides a comprehensive set of neighbors but is not widely used due to its computational complexity induced by the large number of neighbors. In addition, due to the possibility of reaching local optima, the search for the most likely tree usually starts from multiple initial trees. For example, RAxML starts with random trees as well as trees reconstructed based on the maximum parsimony criterion, while IQ-TREE (Nguyen *et al.*, 2015) starts with both maximum parsimony-based trees and Neighbor-Joining-based trees (Saitou and Nei, 1987). Additional suggested search strategies include genetic algorithms (Helaers and Milinkovitch, 2010; Lewis, 1998) and simulated annealing (Stamatakis, 2005). Furthermore, it was previously shown that it is beneficial not to compute the likelihood of neighboring trees whose estimated sum of branch lengths highly deviates from that of the current tree (Hordijk and Gascuel, 2005). We have recently shown that machine-learning algorithms can be efficiently utilized to accurately rank neighboring trees without computing their likelihood, thus potentially increasing the computational efficiency of tree inference (Azouri *et al.*, 2021).

Regardless of the specific search strategy, the most time-consuming operation in ML-based tree search is the evaluation of the log-likelihood of neighboring trees (including branch-length optimization) using the so-called phylogenetic likelihood function. To facilitate efficient likelihood computations, a main assumption of all widely used models is that evolution at different sites is independent, conditioned on the tree. It follows that the likelihood of each site can be computed independently, and that the log-likelihood of a tree is the sum of the per-site log-likelihoods over all MSA sites. Therefore, the running time of the likelihood evaluation on a given tree is theoretically linear in the number of sites in the MSA. However, in practice, programs that perform phylogenetic analysis save computational effort by avoiding repetitive calculations across different sites. For example, one straightforward technique employed by popular programs such as PhyML (Guindon *et al.*, 2010), RAxML (Stamatakis, 2014) or MrBayes (Ronquist *et al.*, 2012) is to remove duplicated columns from the MSA and assign appropriate weights to the remaining unique columns. RAxML-NG (Kozlov *et al.*, 2019) further optimizes the running time by implementing a technique known as site repeats, in which identical site patterns are detected at each subtree, resulting in runtime improvements of 10–60% (Kobert *et al.*, 2017).

Increasing the number of independently evolving loci in an MSA is expected to improve the reliability and robustness of phylogenetic inference (Pamilo and Nei, 1988), although this can also lead to biases in tree inference, e.g. due to discordance between individual gene trees (Degnan and Rosenberg, 2009; Knowles, 2009). In recent years, full-genome sequences have become increasingly available and as a result many modern phylogenetic analyses are based on increasingly longer sequences, often exceeding 100 000 sites (Jarvis *et al.*, 2014; Shen *et al.*, 2020). This trend is expected to become more prominent in the near future with projects such as The Earth BioGenome Project (Lewin *et al.*, 2018) that aims to sequence and assemble the genomes of over 1 000 000 eukaryotic species. The shift toward phylogenetic reconstruction of large-scale MSAs seems promising for increasing inference reliability (Gee, 2003), but at the same time presents a computational challenge to phylogenetic inference methodologies. The common practice of trimming unreliably aligned sites from an MSA prior to phylogenetic reconstruction can potentially contribute to running time reduction. However, it has been argued that on average, trimming an MSA using popular tools has a slightly negative effect on the accuracy of the obtained tree (Tan *et al.*, 2015). Moreover, it has been claimed that this negative effect increases with the number of sites being removed by the trimming process, implying that reducing running time in this manner comes at the cost of reduced accuracy. In light of the issues with current trimming techniques, the ClipKIT tool (Steenwyk *et al.*, 2020) implements a new method for MSA trimming whose strategy is to retain all parsimony-informative sites (i.e. sites that contain at least two characters that appear in at least two taxa)—even if these contain many gaps—and possibly retaining constant sites. Although ClipKIT is claimed to be relatively harmless with respect to tree inference accuracy compared to popular trimming programs, the expected decrease in running-time might be insufficient when working with MSAs with a large proportion of parsimony-informative sites.

Here, we propose a new approach to select a subset of MSA sites as a means to reduce the running time of likelihood-based inference, with minimal reduction in inference accuracy. Our methodology is based on the artificial-intelligence paradigm and utilizes the Lasso-based regression. This approach is based on our observation that for a given MSA, the log-likelihood of different sites across trees is highly correlated. Such correlations can be utilized to identify a representative set of sites and corresponding weights that enable predicting the overall log-likelihood of any given tree.

## 2 New approach

We regard the problem of MSA trimming as a variable-selection task, where each MSA site is considered as a variable and the aim is to choose the smallest set of variables that is sufficient for representing the entire alignment. Our methodology is based on Lasso regression (Tibshirani, 1996), which tends to select only one predictor from any set of highly correlated predictors (Zou and Hastie, 2005). We apply the Lasso regression in order to detect a subset of sites and their associated weights. These sites and weights are used to approximate the log-likelihood of the entire MSA for any given tree. In other words, the Lasso approach provides a sample of sites and a formula by which one can compute the log-likelihood of the entire data based on this sample. Let $LL_{i}\left(T^{j}\right)$ be the log-likelihood of site i given tree $T^{j}$, representing both the tree topology and its associated branch-lengths. ${LL}_{i}\left(T^{j}\right)$ are computed based on a specified Markov substitution model (see Section 3 below), which can be any of the widely used nucleotide or amino-acid replacement models. Assuming independence among MSA sites, the log-likelihood of the MSA given $T^{j}$ satisfies:

(1) $$LL\left(T^{j}\right)=\;\sum_{i=1}^{m}{LL}_{i}(T^{j})$$

where m is the number of sites in the MSA. We next express $LL\left(T^{j}\right)$ as a linear combination of the log-likelihood values of the individual sites $LL_{i}\left(T^{j}\right),1\leq i\leq m$:

(2) $$LL\left(T^{j}\right)=\;\sum_{i=1}^{m}{\beta_{i}LL}_{i}(T^{j})$$

Clearly, setting all beta values to 1 will be optimal in terms of accuracy. However, to reduce computation time we aim to zero some of the beta values, while retaining a good approximation of the likelihood function. Thus, our goal is to find a small set of τ sites (τ≪m) $i_{1},\ldots ,i_{\tau }$, encompassing all sites whose weight is not zero, such that $LL\left(T^{j}\right)\approx \sum_{k=1}^{\tau }\beta_{\left(i_{k}\right)}LL_{i_{k}}\left(T^{j}\right)$.

The detection of the subset of sites is conducted in a training phase, which is performed as an initial analysis for a given dataset. In this training phase, we generate a set of η trees, and for each tree we compute the exact per-site log-likelihoods. These η trees are randomly selected from the set of all possible trees (see section generating random trees in Section 3). The computed log-likelihoods of these trees are provided to the Lasso regressor, which provides the list of τ sites and their associated weights. These sites and weights are then used in the following ML tree-search phase using standard search heuristics. Specifically, the log-likelihoods of the η trees used for training can be expressed in a matrix form:

(3) $$\left(\begin{array}{c} LL(T^{1})\\ \begin{array}{c} LL(T^{2})\\ \vdots \end{array}\\ LL(T^{\eta }) \end{array}\right)=\left(\begin{array}{ccc} {LL}_{1}\left(T^{1}\right) & \cdots & {LL}_{m}\left(T^{1}\right)\\ \vdots & \ddots & \vdots \\ {LL}_{1}\left(T^{\eta }\right) & \cdots & {LL}_{m}\left(T^{\eta }\right) \end{array}\right)\left(\begin{array}{c} \beta_{1}\\ \begin{array}{c} \beta_{2}\\ \vdots \end{array}\\ \beta_{m} \end{array}\right)$$

If one considers the beta vector as unknown, using the least-square approach we search for the beta vector that minimizes:

(4) $${\sum_{j=1}^{\eta }\left((LL\left(T^{j}\right)-\beta_{0}-\sum_{i=1}^{m}\beta_{i}LL_{i}(T^{j})\right)}^{2}$$

$\beta_{0}$ is the intercept of the regression line (the value of $\beta_{0}$ may be ignored during the tree search, as it does not affect the ranking of alternative tree topologies). Setting $\beta_{0}=0$ and $\beta_{1}=\beta_{2}=\cdots \beta_{m}=1$ would make this error expression equal zero. Our goal is to obtain low errors, with fewer than *m* non-zero beta values. To this end, we apply the Lasso criterion (Tibshirani, 1996), namely, we add a penalty term $\sum_{j=1}^{m}\left|\left|\beta_{j}\right|\right|\times \lambda$ where λ is a tuning parameter of the Lasso methodology. We thus aim to minimize:

(5) $${\sum_{j=1}^{\eta }\left((LL\left(T^{j}\right)-\beta_{0}-\sum_{i=1}^{m}\beta_{i}LL_{i}\left(T^{j}\right)\right)}^{2}+\sum_{j=1}^{m}\left|\left|\beta_{j}\right|\right|\times \lambda$$

The higher the value of λ, the fewer the sites that will be selected, i.e. a larger number of beta values will be set to zero. In our case, selecting more sites is expected to improve prediction accuracy. Hence, the value of λ controls the trade-off between prediction accuracy and running time. We thus search for λ values that correspond to a fixed percentage of positions, which we denote by ζ (e.g. 1% or 5% of the sites).

## 3 Materials and methods

### 3.1 Data used for evaluation

In order to evaluate our approach, we used six very large amino-acid MSAs (NagyA1, ShenA9, StruA5, WickA3, YangA8 and MisoA2) from Zhou *et al.* (2018). From each amino-acid MSA, we generated nine datasets by randomly selecting 15, 30 and 60 sequences and trimmed the first 20 000, 40 000 and 80 000 positions, after removing fully undetermined columns from the obtained datasets. Additional analyses were performed on four DNA MSAs (WickD3a, PrumD6, MisoD2a, WickD3b) also from Zhou *et al.* (2018), which were trimmed to 30 taxa and 20 000 positions.

### 3.2 Computing exact site-specific log-likelihoods

The computations of the site-specific log-likelihood values and the log-likelihood value of a given tree based on Lasso weights were performed using RAxML-NG (Kozlov *et al.*, 2019), under the WAG+GAMMA model (Whelan and Goldman, 2001).

### 3.3 Generating a set of random trees

To generate a set of random trees for each dataset, we first generated a set of random topologies $\left(T^{1},.,T^{\eta }\right)$, using the sequential randomized stepwise addition order algorithm, as implemented in RAxML-NG (Kozlov *et al.*, 2019). Branch-lengths were then drawn from an exponential distribution with a mean of 0.1.

### 3.4 Lasso computations

In order to fit a Lasso model for various values of λ, we used the implementation of lasso_path in the Sklearn library (Pedregosa *et al.*, 2011) in Python version 3.8 that generates the Lasso path with coordinate descent.

The grid of penalty parameters is chosen, by default, such that the maximum value in the grid is the minimal penalty which forces all coefficients to equal exactly zero. Here, we used 100 grid points on a log scale such that the ratio between the largest penalty to the minimal penalty equals $1\times 10^{-7}$. In order to find a Lasso solution for a given percentage of non-zero coefficients (ζ), we iterated over the penalty grid until finding a solution matching this criterion. We note that sampling large fractions of the data based on this method is not possible, since this fraction is limited from above by the fraction of the data obtained by the minimal value of the grid. Due to technical and mathematical limitations (RAxML-NG only accepts a site weight vector with positive weights as input), we restricted the Lasso coefficients to be positive. We note that as part of finding the beta vector, the columns of the regression design matrix were normalized by subtracting the column mean and dividing each value by the L2-norm so that selection will be scale-free.

### 3.5 Analyzing the effect of various features on the Lasso performance

The analysis was performed using the *nlme* package (Pinheiro *et al*., 2020) for the R software, version 4.0.3, treating the empirical MSA as a random effect after applying logit transformation on the response variable:

(6) $$y=\log\left(\frac{\left(1-r^{2}\right)}{1-\left(1-r^{2}\right)}\right)=\log\left(\frac{\left(1-r^{2}\right)}{r^{2}}\right)$$

The coefficient of determination ($R^{2}$) was evaluated using the *rsq* package (Zhang, 2021) for the R software, version 2.2, considering fixed effects only.

### 3.6 Evolutionary rate analysis

We used Rate4Site (Pupko *et al.*, 2002) to compute standardized site-specific evolutionary rates. Rate4Site was run with maximum parsimony trees with ML-optimized branch-lengths computed using RAxML-NG. Rates were computed based on the empirical Bayes method.

### 3.7 Tree search algorithm

We implemented a tree-search algorithm, inspired from the speedups introduced in RAxML (Stamatakis *et al.*, 2007). At each iteration, all SPR neighbors of the current tree within a rearrangement distance of five nodes are evaluated (without branch-lengths optimization). Once a better tree is found (a tree with a higher log-likelihood value by at least 0.1 log-likelihood points), the search is immediately repeated starting from that neighbor. In case that none of the proposed neighbors is better than the current tree, full branch-lengths optimization is performed on the 50 best SPR neighbors according to the previous evaluation. Again, once a better tree is found, the search immediately continues from that tree. The search ends when none of the proposed neighbors is better than the current tree.

The Lasso-based tree search was implemented in two phases. When sampling based on the Lasso, instead of computing the log-likelihood based on the entire set of positions, the log-likelihoods were computed based on the positions sampled by the Lasso, multiplying the log-likelihood of each position by its associated weight, which is also given as output by the Lasso approximation. During the first phase, the Lasso-based log-likelihood approximation was used, with the first phase terminating once reaching a local maximum. In the second phase, a second SPR search starts from the final tree of the first phase, while using the Lasso-based log-likelihood approximation for evaluating the log-likelihood of all SPR candidates (without branch-lengths optimization) and using the full MSA to perform branch-lengths optimization on the resulting 50 best trees. The Lasso-based log-likelihood approximation was obtained based on ζ = 5% of the MSA sites, and the training-size (η) was set to 2000 and 4000, for MSAs with 15 sequences and MSAs with either 30 or 60 sequences, respectively.

## 4 Results

### 4.1 Lasso performance evaluation on an example dataset

We first demonstrated the Lasso learning process on the NagyA1 dataset (see Section 3), which was trimmed to have 30 amino-acid sequences and m=80 000 sites. We assumed the WAG amino-acid replacement model (Whelan and Goldman, 2001) with among-site-rate variation modeled by a discrete gamma distribution with four rate categories and an alpha parameter of 0.93, inferred using ML assuming the most parsimonious tree reconstructed by RAxML-NG.

For the training phase, we first generated a training set of per-site log-likelihood values based on η=4000 random tree topologies whose branch-lengths were drawn from an exponential distribution with scale parameter 0.1. For the Lasso modeling, we selected a penalty parameter such that approximately ζ = 5% of the MSA sites (4048 sites) have non-zero coefficients. To evaluate the accuracy of the Lasso approximation on the training data, we compared the full log-likelihoods of the training trees to the log-likelihoods estimated based on the Lasso approximation (note, for these comparisons we did not optimize the branch lengths or any specific model parameters). Table 1A shows the exact and approximated log-likelihoods of five randomly selected trees used for learning. As can be seen, all errors are below 0.009% and the differences between the exact and approximated log-likelihood values are substantially smaller than the differences among the scores of the distinct tree topologies.

**Table 1.** Performance of the Lasso approximation

A.			
Training random tree index	Log-likelihood with site sampling	Log-likelihood with all sites	Percentage of error (%)
1	−2 521 335.5	−2 521 360.5	0.001
2	−2 492 529.8	−2 492 400.3	0.005
3	−2 682 862.6	−2 683 107.8	0.009
4	−2 491 174.8	−2 491 191.2	0.001
5	−2 463 169.9	−2 463 143.8	0.001

B.			
Testing random tree index	Log-likelihood with site sampling	Log-likelihood with all sites	Percentage of error (%)
1	−2 186 578.4	−2 182 258.2	0.198
2	−2 177 328.9	−2 172 830.6	0.207
3	−2 201 897.4	−2 198 910	0.136
4	−2 137 834.4	−2 132 177.7	0.265
5	−2 174 970	−2 170 486.8	0.207


*Note*: Performance of the Lasso approximation on five trees selected from the training set (A) and five trees selected from the test set (B). The training and test sets included 4000 and 100 trees, respectively. The Lasso methodology selected 4048 sites from a total of 80 000 sites (i.e. around 5%). The percentage of error is calculated as the absolute of the difference between the true and approximated log-likelihoods divided by the true log-likelihood. The mean percentage of error and the standard deviation across 4000 trees used as a training set are 0.0035 and 7.6e-06, respectively. The mean percentage of error and the standard deviation across 100 trees used as a test set are 0.17 and 0.0019, respectively.

**Fig. 1.**
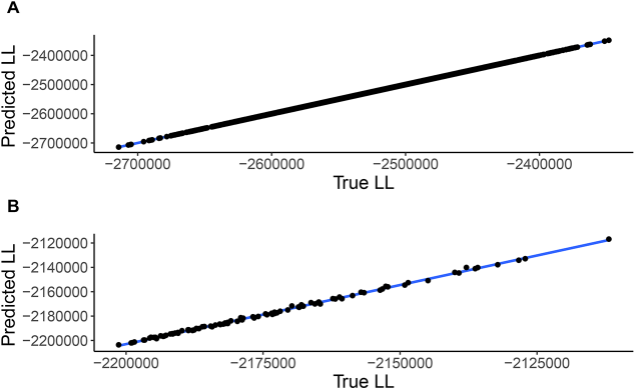
Scatter plot of predicted versus exact log-likelihoods (LL). Each dot represents one random tree. The blue line is the linear regression line. (**A**) Results on training data; (**B**) results on test data

We then tested the performance of the approximation on 100 additional random trees that had not been used for training the model (test data). While the branch lengths of the trees used for training were drawn from an exponential distribution, the branch-lengths of the trees used for testing were ML estimated based on all MSA positions. Table 1B shows the same comparison as above, for five randomly selected trees from these test data (full results are available in Supplementary Table S1). To better quantify the performance, we computed the squared correlation coefficient (Pearson $r^{2}$) between the approximated and the exact log-likelihoods (Fig. 1) and observed very high performance on the training and test datasets ($r^{2}$ > 0.999 for training set and $r^{2}$ = 0.998 on the test set). Moreover, the relative order of the log-likelihoods of the examined trees with the Lasso approximation was nearly identical to that with the full set of MSA positions (Spearman ρ > 0.999 on the training set, ρ = 0.998 on the test set).

**Fig. 2.**
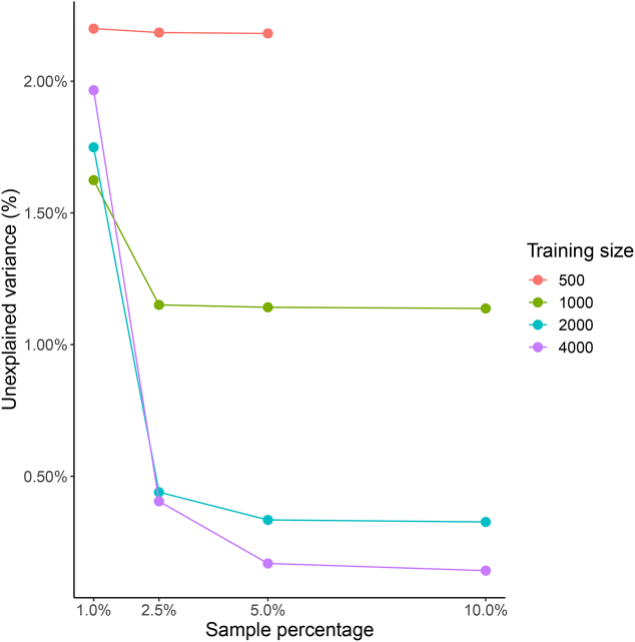
The error in log-likelihood estimation as a function of training size and percentage of sampled positions. The *y* axis quantifies the error as the percentage of unexplained variance ($1-r^{2}$) obtained on a test set of 100 random trees ($r^{2}$ denotes the square Pearson correlation coefficient). Shown are results for four values of training size and four values of sampling percentage. The analyzed alignment is the NagyA1 dataset with 30 sequences and 80 000 sites

The above results were obtained when the Lasso procedure was trained on η = 4000 random trees and setting the percentage of sampled positions (ζ) to 5%. In order to estimate the effect of both η and ζ on prediction accuracy, we trained the Lasso model several times based on different combinations of η (500, 1000, 2000, 4000) and ζ (1%, 2.5%, 5%, 10%), and assessed the respective accuracy on the test set. As expected, the best result is obtained when using the largest training size (η = 4000) and the largest sample percentage (ζ = 10%), with $r^{2}$ = 0.9985 (Fig. 2). However, using the same training size (η = 4000) and only ζ = 5% of the positions provides very close accuracy ($r^{2}$ = 0.9983). In addition, the overall performance of training sizes 2000 and 4000 along the different sample percentages was relatively similar. For the smallest sample percentage (ζ = 1%) having larger training-size did not improve test accuracy. However, with increasing sample percentage the advantage of having a larger training size became substantial (Fig. 2). To gain further insights into the robustness of the Lasso-based sampling approach, we followed the same procedure as described above, running on 54 different alignments: six different empirical MSAs, which were randomly trimmed to include 15, 30 or 60 sequences, with alignment lengths of either 20 000, 40 000 or 80 000 positions. For each of these 54 datasets, we tested the accuracy as a function of four values of η: 500, 1000, 2000, 4000, and four ζ values: 1%, 2.5%, 5%, 10%. The prediction accuracy was evaluated on a random sample of 100 trees, whose branch-lengths were ML-optimized using the full MSA. In most cases, the error rates were lower than 5%, and the increase in accuracy when moving from ζ = 5% to ζ = 10% was negligible (full results are available in Supplementary Table S2, Supplementary Fig. S1).

### 4.2 Factors affecting the Lasso performance

We used a linear mixed-effect model to describe the relationship between the unexplained variance on the test set ($1-r^{2}$) and the following explanatory variables: sequence divergence, percentage of constant sites in the MSA, the alpha parameter of the gamma distribution, number of sequences and alignment length (see Section 3 for details). Our analysis obtained an $R^{2}$ of 0.86 and suggested that the most important factors affecting accuracy are the MSA length, the number of sequences and the alpha parameter: both longer alignments and larger alpha parameters (less rate heterogeneity among sites) lead to reduced errors while increasing the number of sequences increases the error. Specifically, adding ten sequences to the MSA has an opposite effect on the error as adding 10 000 new sites or increasing the alpha parameter by 0.25. Sequence divergence and percentage of invariant sites had a non-significant effect on accuracy (full results are available in Supplementary Table S3).

### 4.3 Lasso performance under other evolutionary models

The above results were obtained under the WAG replacement matrix. To test the performance of our approach under alternative protein evolutionary models, we applied the Lasso procedure (using η=4000 random trees) under the JTT and LG models on the above six empirical amino-acid MSAs trimmed to 20 000 positions and 30 taxa. The absolute difference in accuracy (measure by $r^{2}$ between the true and inferred log-likelihoods) using WAG and using either JTT or LG were always lower than 0.016, suggesting that the Lasso approach is insensitive to the choice of the protein model. We also tested the performance of the Lasso procedure on four DNA alignments by applying the Lasso procedure assuming the GTR+G model (using η=4000, 20 000 positions, 30 taxa). The accuracy was still very high (mean $r^{2}$ = 0.97 using ζ=5% of the positions), albeit slightly inferior to that obtained when analyzing the protein datasets (full results for all protein and DNA models are available in Supplementary Table S4).

### 4.4 The evolutionary rate of sampled positions

**Fig. 3.**
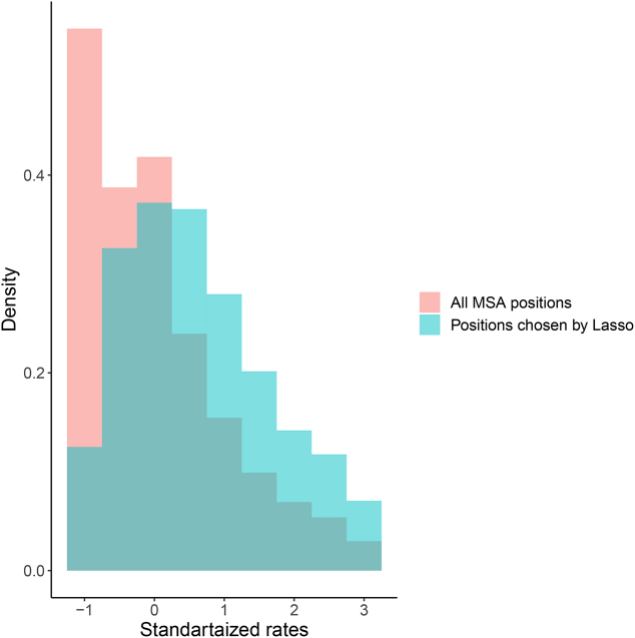
Distribution of evolutionary rates for the entire alignment against that of the sampled alignment. Shown are results on the NagyA1 dataset with 30 sequences and 80 000 positions using ζ = 5% of the positions and η  *=* 4 000 trees used for training. The overlap between the two distribution is shown in dark green

We hypothesized that the positions selected by the Lasso are not a random sample of all MSA positions. To test this hypothesis, we next quantified the site-specific evolutionary rates of all MSA positions (See Section 3). Figure 3 shows the distributions of evolutionary rates for the entire alignment against that of the sampled positions for the NagyA1 dataset with 30 sequences and 80 000 positions. This comparison suggests that the Lasso algorithm tends to select positions with relatively high evolutionary rate: while the average rate across all positions is normalized to have a mean of zero, the average rate of selected positions was 0.61 (*P* < 2.2E–16; *t*-test). This suggests that the Lasso approximation relies on the more variable sites, which are phylogenetically informative, on account of the more conserved sites, which are highly correlated to each other. Similar results were obtained for other datasets and other combinations of ζ and η (see Supplementary Fig. S2).

### 4.5 Applying the Lasso approximation to partitioned data

The above results were obtained under the assumption of a single evolutionary model for the entire MSA. However, different genes may differ in their evolutionary model. To demonstrate the utility of the Lasso approach under such a scenario, we analyzed the performance on a partitioned data. Specifically, three empirical datasets (NagyA1, YangA8 and PrumD6, each dataset trimmed to 30 sequences and 20 000 positions) were used, whereas in each dataset, at least 12 gene regions (partitions) exist. The Lasso parameters used were η=4000 and ζ=5%. We compared the performance of applying the Lasso procedure once for the concatenated alignment versus applying it to each partition separately. In such a partition analysis we first fitted a separate model for each gene partition and then generated training data, such that the log-likelihoods of each position are computed based on its corresponding model. The Lasso procedure was then applied to these training data. The accuracy of the test sets using the concatenation and partition approaches was very similar for all three datasets (absolute difference in test set accuracy smaller than 0.0015), suggesting that the Lasso approach can accurately account for model variation among different partitions.

We next examined whether the Lasso methodology tends to select more positions from fast-evolving genes. First, we observed that the sampled positions are not distributed as expected if no bias exists, i.e. in some genes more than 5% of the positions were sampled, while in others, less (*P*-value < 1.2 × 10^−11^; Chi-square). Rather, for all three datasets higher sampling percentages were observed for fast-evolving genes. Specifically, a significant correlation was found between the mean evolutionary rate of each gene and the sampling fraction (Pearson *r *=* *0.17, 0.73 and 0.14 for the NagyA1, Yang8, PrumD6 datasets, respectively when using a concatenated dataset and *r *=* *0.57. 0.87, 0.33 for the NagyA1, Yang8, PrumD6 datasets, respectively when using partitioned data).

Although the above results suggest a bias sampling of the Lasso approach, favoring positions from fast-evolving genes, we next tested whether this oversampling affects the ranking of tree topologies. Specifically, it is possible that any sample of positions from the entire dataset would bias the ranking of competing tree topologies. If we observe that the Lasso approximation, which is based on all partitions, overestimates the log-likelihood of some trees and these same trees are also overestimated when using only the fast-evolving genes, we can conclude that the Lasso approximation is biased toward the fast-evolving genes. If, however, possible biases in tree ranking introduced by the fast-evolving partitions have a small impact on the Lasso approximation, we expect a low, if any association between the trees over-estimated by the fast-evolving genes and trees over-estimated by the Lasso approximation. To determine if such a bias exists and if so—its magnitude, for each dataset, we selected the top three fast-evolving genes and used them to evaluate the log likelihood of 100 test trees. To determine the bias in log-likelihood estimate we computed the difference between the estimated and true log-likelihood for each tree. We repeated this bias computation, this time applying the Lasso approach for all genes. We then computed the correlation between the errors. No significant positive correlation was observed, suggesting that the fast-evolving trees and the Lasso approximation do not significantly overestimate and underestimate the log likelihoods of the same trees. This suggests that the impact of the fast-evolving trees on the selection of tree topologies is relatively small, despite the fact that more positions are sampled from these genes.

### 4.6 Comparison of the Lasso procedure to naïve approaches

We compared the performance of the Lasso procedure (using η=4000 random trees) to two alternative naïve procedures: (i) sampling positions randomly (results were averaged over five iterations); (ii) Selecting the positions with the highest evolutionary rate. We performed this comparison on the six empirical amino-acid MSAs, trimmed to 20 000 positions and 30 sequences, using a test set of 100 random trees. Our results indicated that random sampling is superior to the sampling of the fastest evolving positions and that both approaches are inferior to the Lasso approximation, particularly when the sampling fraction is low. For example, when 10% of the positions were sampled, the mean of $r^{2}$ were 0.82, 0.97 and 0.99 for the sampling of fast positions, random sampling and Lasso-based sampling, respectively. These values were 0.57, 0.79 and 0.92, when using ζ=1%. Full results are available in Supplementary Table S5.

### 4.7 Lasso performance during an SPR search on the example dataset

We next evaluated the benefit of using this approximation in a hill-climbing heuristic for finding the ML-tree topology, starting from a random tree. To this end, we implemented three variants of a greedy tree-search heuristic: (i) standard: without using Lasso; (ii) Lasso-only; (iii) two-phase search, in which an additional search phase initiates from the ending point of the Lasso-only search, but this time the branch lengths of proposed neighbors are optimized using all alignment positions (see Section 3 for details). The performance of the three search strategies on an example MSA (NagyA1), initiating from the same starting tree (with a log-likelihood of -2 191 145.5) are summarized in Table 2. The results indicate that the Lasso-only search (without accounting for the training time) is 47-fold faster than the standard search, albeit it led to a slightly inferior tree topology. Adding the second search phase resulted in a tree that had a slightly higher likelihood than the tree obtained using the standard search, with a 22-fold reduction in running time. Most of the gain in log-likelihood was obtained during the first phase (Supplementary Fig. S3). The second phase of the algorithm, which is computationally demanding was required to distinguish between tree topologies that are near the locally optimal tree. For this distinction, accurate estimation of branch lengths based on all MSA positions is needed.

**Table 2.** Performance of different search strategies

	Standard	Lasso-only	Two-phase
Log-likelihood of final tree	−1 925 986.1	−1 926 169.6	−1 925 950.8
Number of SPR moves	167	153	157
Total CPU time of the search	133 818	2847	6208
Training CPU time	0	16 502	16 502

*Note*: Performance of the Standard search, Lasso-only search and Two-phase search on the NagyA1 MSA with 30 sequences and 80 000 positions. The Lasso-only search and the Two-phase search are based on Lasso approximation, which was generated using ζ = 5% of the positions and η  *=* 4000 trees for training. All final log-likelihood scores are computed using all alignment sites.

In both the Lasso-only and the two-phase searches, the Lasso weights must be computed prior to running the tree-search algorithm. The above results are based on a single starting tree. However, state-of-the-art heuristics start with multiple starting trees, e.g. in RAxML-NG the default number of random starting trees is 10. Notably, the training phase of the Lasso approximation needs only to be conducted once for all starting trees. Thus, assuming similar computational times for 10 searches starting from different random points, the two-phase algorithm is expected to be 17.02 times faster than the standard search, when accounting for the training time. When a single starting tree is used, the two-phase algorithm is 5.9 times faster than the standard search (Table 2).

### 4.8 Lasso performance during the SPR search on additional datasets

We followed the same pipeline on 18 empirical MSAs, each with a total of 80 000 positions (six amino-acid datasets, trimmed to include either 15, 30 or 60 sequences), starting the searches from three random points. For 15 sequences, the two-phase algorithm converged to a tree with higher log-likelihood compared to the standard algorithm for one out of the six datasets, and to the same ML trees for the remaining five datasets. The median fold decrease in computational time of the two-phases algorithm was 3.72. This modest running-time factor is due to the relative cost of the training time compared to the small number of SPR moves required to reach a local maximum for such a small search space. For MSAs with 30 and 60 species, the running time improvement was substantially higher (median fold decrease in running time of 7.94 and 19.24, for 30 and 60 sequences, respectively). The accuracy of the two algorithms is comparable, i.e. out of the twelve empirical datasets with either 30 or 60 sequences, in five of the cases, the two-phase algorithm converged to a tree with higher log-likelihood, in four cases it converged to a tree with a lower log-likelihood and in the remaining three cases the two algorithms converged to the same tree (full results are in Supplementary Table S6).

## 5 Discussion

In this work, we presented a numerical approach to reduce the computation time required to evaluate the log-likelihood of a phylogenetic tree with respect to a given MSA. The underlying assumption in our approach is that information contained in many of the MSA sites, especially when large MSA are used, is highly redundant. Thus, inference based on a small number of sites, when chosen carefully, can greatly reduce running times, while maintaining inference accuracy. Applying this approach on several empirical MSAs, we have demonstrated that in all cases, using the proposed 5% of the MSA positions is sufficient to obtain a good approximation of true log-likelihood values, i.e. log-likelihood values calculated using the entire MSA. Furthermore, we have shown that using our approximation during SPR searches can substantially reduce running-time with little to none effect on inference accuracy.

In our analysis, we have used a fixed percentage of sampled position (ζ) to each empirical dataset and throughout the entire search. A possible extension of the work presented here would be to fit ζ to each dataset, based on its characteristics, e.g. the alignment length, number of sequences and the extent of among-site rate variability. Furthermore, it may be beneficial to dynamically adjust ζ as the search progresses, i.e. use very low ζ values for the first steps of the tree search, and slowly increase the value as we approach local maxima. Similarly, we have used a fixed number of random trees for training (η) for all datasets. However, it is possible that learning based on a selected set of non-random trees may prove to be more beneficial, e.g. bootstrap trees. Moreover, it is possible that multiple training of the Lasso model along the tree search may be beneficial, e.g. re-training the Lasso based on near-optimal trees in advance stages of the tree search. Finally, it is possible to train a learning algorithm such as neural network to automatically detect the best hyper-parameter tuning strategy for a given empirical dataset, based on a large number of training datasets. We showed that our approach can be extended to mixed/partitioned analysis simply by applying it separately on each part of the MSA and sum the corresponding results to obtain an approximation for the entire MSA. While the application of this approach to codon MSAs is straightforward, it is important to test how different data characteristics and hyperparameters affect performance on this type of data. The Lasso approach was also recently used in order to select a subset of informative loci for phylogenetic analysis (Kumar and Sharma, 2021). In that method the regression model did not aim at reducing running times nor to compute the log-likelihood of alternative trees. When very large datasets composed of multiple loci are analyzed, these two approaches can potentially be combined to even further reduce running time by first selecting a small set of loci, and subsequently selecting a small set of positions within each locus. Finally, the proposed methodology can potentially reduce the time of Bayesian approaches in which the log-likelihood computations is a major time-consuming step.

We note that the Lasso approximation should be valuable also when other tree-search algorithms are used, e.g. when Nearest Neighbor Interchange (NNI) moves are used instead of SPR or in cases were approximate post-SPR log-likelihoods are computed using local branch-lengths optimization, as is done, for example, in PhyML (Guindon *et al.*, 2010) and RAxML-NG (Kozlov *et al.*, 2019).

## Funding

N.E. and D.A. were supported in part by a fellowship from the Edmond J. Safra Center for Bioinformatics at Tel Aviv University. D.A. was supported by The Council for Higher Education program for excellent Ph.D. students in Data Sciences and by a fellowship from the Fast and Direct Ph.D. Program at Tel Aviv University. Y.M. was supported in part by a grant of the Israel Science Foundation (ISF) 993/17. T.P. was supported by Israel Science Foundation grants 802/16 and 2818/21.

*Conflict of Interest*: none declared.

## Supplementary Material

btac252_Supplementary_Data
