# Supplementary material for: A LASSO-based approach to sample sites for phylogenetic tree search
Source: Bioinformatics. 2022 Jun 27;38(Suppl 1):i118–24. doi: 10.1093/bioinformatics/btac252 (PMC9236582; doi:10.1093/bioinformatics/btac252)
Supplement: btac252_Supplementary_Data [file btac252_supplementary_data.docx]

**A LASSO-based approach to sample sites for phylogenetic tree search**

**Supplementary information**

Noa Ecker^1^, Dana Azouri^1,2^, Ben Bettisworth^3,4^, Alexandros Stamatakis^3,4^, Yishay Mansour^5^, Itay Mayrose^2†^, Tal Pupko^1†^

^1^ [The Shmunis School of Biomedicine and Cancer Research](https://en-lifesci.tau.ac.il/lp-en-mcbb), George S. Wise Faculty of Life Sciences, Tel Aviv University, Tel Aviv 69978, Israel.

^2^ School of Plant Sciences and Food Security, George S. Wise Faculty of Life Sciences, Tel Aviv University, Tel Aviv 69978, Israel.

^3^Computational Molecular Evolution Group, Heidelberg Institute for Theoretical Studies, 69118, Heidelberg, Germany

^4^ Department of Informatics, Institute of Theoretical Informatics, Karlsruhe Institute of Technology, 76128 Karlsruhe, Germany.

^5^ The Blavatnik School of Computer Science, Raymond & Beverly Sackler Faculty of Exact Sciences, Tel Aviv University, Tel Aviv 69978, Israel.

† To whom correspondence should be addressed:

Tal Pupko, Tel: +972 3 640 7693; Fax: +972 3 642 2046; E-mail: [talp@tauex.tau.ac.il](mailto:talp@tauex.tau.ac.il)

Itay Mayrose, Tel: +972 3 640 7212; Fax: +972 3 640 9380; E-mail: [itaymay@tauex.tau.ac.il](mailto:itaymay@tauex.tau.ac.il)

Keywords: molecular evolution, phylogenetic tree search, Lasso regression, site sampling

# **Table S1.**

Log-likelihood predictions of the Lasso approximation on the test set of 100 random trees. The Lasso methodology selected 4,048 sites from a total of 80,000 sites (i.e., around 5%)

| **Test random tree index** | **Log-likelihood with site sampling** | **Log-likelihood with all sites** |
| --- | --- | --- |
| 1 | -2,186,578.35 | -2,182,258.22 |
| 2 | -2,177,328.91 | -2,172,830.63 |
| 3 | -2,201,897.38 | -2,198,909.96 |
| 4 | -2,137,834.36 | -2,132,177.74 |
| 5 | -2,174,969.99 | -2,170,486.76 |
| 6 | -2,132,907.25 | -2,127,155.17 |
| 7 | -2,186,566.92 | -2,182,914.62 |
| 8 | -2,188,243.53 | -2,184,566.09 |
| 9 | -2,191,425.05 | -2,187,588.13 |
| 10 | -2,202,025.12 | -2,199,077.42 |
| 11 | -2,182,779.45 | -2,179,054.11 |
| 12 | -2,190,134.99 | -2,187,179.63 |
| 13 | -2,181,713.03 | -2,178,645.04 |
| 14 | -2,172,812.21 | -2,168,418.34 |
| 15 | -2,194,671.41 | -2,191,913.71 |
| 16 | -2,191,838.98 | -2,188,633.80 |
| 17 | -2,116,822.30 | -2,111,888.92 |
| 18 | -2,195,809.14 | -2,192,736.44 |
| 19 | -2,165,674.53 | -2,162,266.47 |
| 20 | -2,170,757.15 | -2,168,005.20 |
| 21 | -2,197,401.66 | -2,195,472.75 |
| 22 | -2,165,952.38 | -2,161,821.41 |
| 23 | -2,177,301.59 | -2,172,457.54 |
| 24 | -2,203,603.96 | -2,201,358.35 |
| 25 | -2,188,569.65 | -2,185,706.92 |
| 26 | -2,180,589.70 | -2,176,850.95 |
| 27 | -2,157,771.61 | -2,153,234.58 |
| 28 | -2,183,891.36 | -2,180,866.86 |
| 29 | -2,163,174.64 | -2,158,674.97 |
| 30 | -2,194,388.61 | -2,191,525.95 |
| 31 | -2,193,925.01 | -2,191,032.75 |
| 32 | -2,178,190.27 | -2,174,545.79 |
| 33 | -2,160,399.55 | -2,157,121.06 |
| 34 | -2,190,285.58 | -2,186,713.46 |
| 35 | -2,185,632.05 | -2,181,908.86 |
| 36 | -2,155,984.72 | -2,152,226.23 |
| 37 | -2,191,825.89 | -2,187,873.90 |
| 38 | -2,194,079.11 | -2,190,499.70 |
| 39 | -2,168,467.66 | -2,164,828.26 |
| 40 | -2,186,076.33 | -2,181,440.61 |
| 41 | -2,170,011.46 | -2,165,543.90 |
| 42 | -2,183,114.02 | -2,178,821.80 |
| 43 | -2,170,047.42 | -2,164,552.59 |
| 44 | -2,140,169.39 | -2,135,822.30 |
| 45 | -2,160,792.15 | -2,156,569.88 |
| 46 | -2,188,761.80 | -2,184,308.00 |
| 47 | -2,199,692.28 | -2,196,799.92 |
| 48 | -2,177,692.15 | -2,173,437.19 |
| 49 | -2,188,755.56 | -2,186,077.13 |
| 50 | -2,198,038.64 | -2,195,697.43 |
| 51 | -2,178,593.85 | -2,173,300.03 |
| 52 | -2,187,371.84 | -2,183,702.53 |
| 53 | -2,185,160.66 | -2,181,434.03 |
| 54 | -2,164,362.77 | -2,161,053.94 |
| 55 | -2,186,981.92 | -2,182,808.29 |
| 56 | -2,198,526.36 | -2,194,443.92 |
| 57 | -2,158,719.98 | -2,153,646.57 |
| 58 | -2,177,842.35 | -2,172,861.28 |
| 59 | -2,140,112.72 | -2,137,899.39 |
| 60 | -2,180,916.05 | -2,176,684.39 |
| 61 | -2,171,858.48 | -2,167,658.42 |
| 62 | -2,201,264.40 | -2,198,479.20 |
| 63 | -2,194,666.20 | -2,191,490.57 |
| 64 | -2,196,492.32 | -2,193,077.82 |
| 65 | -2,181,310.86 | -2,179,076.40 |
| 66 | -2,196,204.80 | -2,193,696.21 |
| 67 | -2,197,443.56 | -2,194,658.99 |
| 68 | -2,176,018.93 | -2,171,880.69 |
| 69 | -2,184,265.64 | -2,179,706.10 |
| 70 | -2,184,312.07 | -2,179,692.21 |
| 71 | -2,150,857.86 | -2,144,904.88 |
| 72 | -2,196,628.96 | -2,193,231.35 |
| 73 | -2,191,245.62 | -2,188,697.24 |
| 74 | -2,180,317.96 | -2,175,880.44 |
| 75 | -2,144,622.62 | -2,139,251.35 |
| 76 | -2,191,591.50 | -2,187,794.36 |
| 77 | -2,191,270.27 | -2,188,554.93 |
| 78 | -2,182,448.48 | -2,179,081.11 |
| 79 | -2,134,157.91 | -2,128,332.09 |
| 80 | -2,199,623.31 | -2,196,672.28 |
| 81 | -2,181,563.40 | -2,176,628.42 |
| 82 | -2,185,600.92 | -2,181,489.16 |
| 83 | -2,169,004.03 | -2,164,981.68 |
| 84 | -2,197,512.01 | -2,194,936.36 |
| 85 | -2,155,582.38 | -2,152,613.55 |
| 86 | -2,144,145.32 | -2,140,005.85 |
| 87 | -2,178,878.69 | -2,174,284.07 |
| 88 | -2,141,081.10 | -2,136,287.37 |
| 89 | -2,152,570.45 | -2,148,520.77 |
| 90 | -2,154,538.34 | -2,149,139.03 |
| 91 | -2,165,751.69 | -2,160,604.77 |
| 92 | -2,191,980.90 | -2,189,801.25 |
| 93 | -2,171,776.47 | -2,167,962.47 |
| 94 | -2,193,439.31 | -2,190,527.94 |
| 95 | -2,176,828.76 | -2,172,905.54 |
| 96 | -2,186,098.67 | -2,181,896.27 |
| 97 | -2,171,673.99 | -2,169,739.06 |
| 98 | -2,168,939.92 | -2,166,248.52 |
| 99 | -2,172,503.45 | -2,168,545.21 |
| 100 | -2,199,699.14 | -2,196,611.04 |

# **Table S2.**

Each of the six panels corresponds to a different empirical MSA. Shown is the square Pearson correlation coefficient ($r^{2}$) obtained on a test set of 100 random trees for four three values of alignment length, three values of number of sequences, four values of training size and four values of sampling percentage.

## **MisoA2 dataset**

| **Number of sequences** | **Number of positions** | **Training size** | **Sample percentage** | **Number of positions** | **Coefficient of determination (**$\boldsymbol{r}^{\boldsymbol{2}}$**)** |
| --- | --- | --- | --- | --- | --- |
| 15 | 20,000 | 500 | 1.0% | 214 | 0.948 |
| 15 | 20,000 | 500 | 2.5% | 508 | 0.987 |
| 15 | 20,000 | 500 | 5.0% | 1,043 | 0.989 |
| 15 | 20,000 | 500 | 10.0% | 2,122 | 0.989 |
| 15 | 20,000 | 1,000 | 1.0% | 221 | 0.947 |
| 15 | 20,000 | 1,000 | 2.5% | 516 | 0.985 |
| 15 | 20,000 | 1,000 | 5.0% | 1,035 | 0.995 |
| 15 | 20,000 | 1,000 | 10.0% | 2,060 | 0.997 |
| 15 | 20,000 | 2,000 | 1.0% | 213 | 0.938 |
| 15 | 20,000 | 2,000 | 2.5% | 544 | 0.983 |
| 15 | 20,000 | 2,000 | 5.0% | 1,008 | 0.993 |
| 15 | 20,000 | 2,000 | 10.0% | 2,115 | 0.998 |
| 15 | 20,000 | 4,000 | 1.0% | 226 | 0.948 |
| 15 | 20,000 | 4,000 | 2.5% | 530 | 0.983 |
| 15 | 20,000 | 4,000 | 5.0% | 1,070 | 0.993 |
| 15 | 20,000 | 4,000 | 10.0% | 2,063 | 0.998 |
| 15 | 40,000 | 500 | 1.0% | 400 | 0.971 |
| 15 | 40,000 | 500 | 2.5% | 1,034 | 0.981 |
| 15 | 40,000 | 500 | 5.0% | 2,112 | 0.982 |
| 15 | 40,000 | 500 | 10.0% | 4,052 | 0.982 |
| 15 | 40,000 | 1,000 | 1.0% | 410 | 0.968 |
| 15 | 40,000 | 1,000 | 2.5% | 1,018 | 0.994 |
| 15 | 40,000 | 1,000 | 5.0% | 2,103 | 0.996 |
| 15 | 40,000 | 1,000 | 10.0% | 4,159 | 0.996 |
| 15 | 40,000 | 2,000 | 1.0% | 405 | 0.967 |
| 15 | 40,000 | 2,000 | 2.5% | 1,041 | 0.992 |
| 15 | 40,000 | 2,000 | 5.0% | 2,042 | 0.997 |
| 15 | 40,000 | 2,000 | 10.0% | 4,014 | 0.999 |
| 15 | 40,000 | 4,000 | 1.0% | 425 | 0.972 |
| 15 | 40,000 | 4,000 | 2.5% | 1,053 | 0.992 |
| 15 | 40,000 | 4,000 | 5.0% | 2,125 | 0.998 |
| 15 | 40,000 | 4,000 | 10.0% | 4,140 | 0.999 |
| 15 | 80,000 | 500 | 1.0% | 849 | 0.978 |
| 15 | 80,000 | 500 | 2.5% | 2,145 | 0.980 |
| 15 | 80,000 | 500 | 5.0% | 4,162 | 0.981 |
| 15 | 80,000 | 500 | 10.0% | 8,294 | 0.981 |
| 15 | 80,000 | 1,000 | 1.0% | 803 | 0.984 |
| 15 | 80,000 | 1,000 | 2.5% | 2,039 | 0.994 |
| 15 | 80,000 | 1,000 | 5.0% | 4,284 | 0.995 |
| 15 | 80,000 | 1,000 | 10.0% | 8,224 | 0.995 |
| 15 | 80,000 | 2,000 | 1.0% | 808 | 0.984 |
| 15 | 80,000 | 2,000 | 2.5% | 2,052 | 0.996 |
| 15 | 80,000 | 2,000 | 5.0% | 4,092 | 0.998 |
| 15 | 80,000 | 2,000 | 10.0% | 8,169 | 0.998 |
| 15 | 80,000 | 4,000 | 1.0% | 823 | 0.982 |
| 15 | 80,000 | 4,000 | 2.5% | 2,135 | 0.997 |
| 15 | 80,000 | 4,000 | 5.0% | 4,029 | 0.999 |
| 15 | 80,000 | 4,000 | 10.0% | 8,077 | 0.999 |
| 30 | 20,000 | 500 | 1.0% | 208 | 0.914 |
| 30 | 20,000 | 500 | 2.5% | 501 | 0.957 |
| 30 | 20,000 | 500 | 5.0% | 1,087 | 0.961 |
| 30 | 20,000 | 500 | 10.0% | 2,094 | 0.962 |
| 30 | 20,000 | 1,000 | 1.0% | 200 | 0.922 |
| 30 | 20,000 | 1,000 | 2.5% | 500 | 0.950 |
| 30 | 20,000 | 1,000 | 5.0% | 1,005 | 0.981 |
| 30 | 20,000 | 1,000 | 10.0% | 2,017 | 0.984 |
| 30 | 20,000 | 2,000 | 1.0% | 216 | 0.926 |
| 30 | 20,000 | 2,000 | 2.5% | 525 | 0.949 |
| 30 | 20,000 | 2,000 | 5.0% | 1,035 | 0.981 |
| 30 | 20,000 | 2,000 | 10.0% | 2,001 | 0.995 |
| 30 | 20,000 | 4,000 | 1.0% | 212 | 0.927 |
| 30 | 20,000 | 4,000 | 2.5% | 543 | 0.942 |
| 30 | 20,000 | 4,000 | 5.0% | 1,005 | 0.971 |
| 30 | 20,000 | 4,000 | 10.0% | 2,019 | 0.992 |
| 30 | 40,000 | 500 | 1.0% | 408 | 0.906 |
| 30 | 40,000 | 500 | 2.5% | 1,019 | 0.932 |
| 30 | 40,000 | 500 | 5.0% | 2,077 | 0.933 |
| 30 | 40,000 | 500 | 10.0% | 4,018 | 0.933 |
| 30 | 40,000 | 1,000 | 1.0% | 412 | 0.900 |
| 30 | 40,000 | 1,000 | 2.5% | 1,013 | 0.969 |
| 30 | 40,000 | 1,000 | 5.0% | 2,016 | 0.974 |
| 30 | 40,000 | 1,000 | 10.0% | 4,053 | 0.974 |
| 30 | 40,000 | 2,000 | 1.0% | 407 | 0.908 |
| 30 | 40,000 | 2,000 | 2.5% | 1,025 | 0.968 |
| 30 | 40,000 | 2,000 | 5.0% | 2,018 | 0.992 |
| 30 | 40,000 | 2,000 | 10.0% | 4,120 | 0.994 |
| 30 | 40,000 | 4,000 | 1.0% | 428 | 0.911 |
| 30 | 40,000 | 4,000 | 2.5% | 1,004 | 0.965 |
| 30 | 40,000 | 4,000 | 5.0% | 2,033 | 0.991 |
| 30 | 40,000 | 4,000 | 10.0% | 4,082 | 0.998 |
| 30 | 80,000 | 500 | 1.0% | 843 | 0.916 |
| 30 | 80,000 | 500 | 2.5% | 2,072 | 0.916 |
| 30 | 80,000 | 500 | 5.0% | 4,028 | 0.916 |
| 30 | 80,000 | 1,000 | 1.0% | 808 | 0.956 |
| 30 | 80,000 | 1,000 | 2.5% | 2,071 | 0.974 |
| 30 | 80,000 | 1,000 | 5.0% | 4,077 | 0.975 |
| 30 | 80,000 | 1,000 | 10.0% | 8,216 | 0.975 |
| 30 | 80,000 | 2,000 | 1.0% | 839 | 0.958 |
| 30 | 80,000 | 2,000 | 2.5% | 2,016 | 0.991 |
| 30 | 80,000 | 2,000 | 5.0% | 4,141 | 0.992 |
| 30 | 80,000 | 2,000 | 10.0% | 8,511 | 0.992 |
| 30 | 80,000 | 4,000 | 1.0% | 869 | 0.951 |
| 30 | 80,000 | 4,000 | 2.5% | 2,074 | 0.988 |
| 30 | 80,000 | 4,000 | 5.0% | 4,006 | 0.996 |
| 30 | 80,000 | 4,000 | 10.0% | 8,650 | 0.997 |
| 60 | 20,000 | 500 | 1.0% | 216 | 0.855 |
| 60 | 20,000 | 500 | 2.5% | 506 | 0.954 |
| 60 | 20,000 | 500 | 5.0% | 1,010 | 0.956 |
| 60 | 20,000 | 500 | 10.0% | 2,033 | 0.956 |
| 60 | 20,000 | 1,000 | 1.0% | 229 | 0.809 |
| 60 | 20,000 | 1,000 | 2.5% | 526 | 0.874 |
| 60 | 20,000 | 1,000 | 5.0% | 1,004 | 0.969 |
| 60 | 20,000 | 1,000 | 10.0% | 2,042 | 0.971 |
| 60 | 20,000 | 2,000 | 1.0% | 211 | 0.866 |
| 60 | 20,000 | 2,000 | 2.5% | 515 | 0.921 |
| 60 | 20,000 | 2,000 | 5.0% | 1,033 | 0.968 |
| 60 | 20,000 | 2,000 | 10.0% | 2,022 | 0.991 |
| 60 | 20,000 | 4,000 | 1.0% | 221 | 0.881 |
| 60 | 20,000 | 4,000 | 2.5% | 535 | 0.920 |
| 60 | 20,000 | 4,000 | 5.0% | 1,022 | 0.962 |
| 60 | 20,000 | 4,000 | 10.0% | 2,085 | 0.992 |
| 60 | 40,000 | 500 | 1.0% | 400 | 0.927 |
| 60 | 40,000 | 500 | 2.5% | 1,034 | 0.940 |
| 60 | 40,000 | 500 | 5.0% | 2,030 | 0.940 |
| 60 | 40,000 | 1,000 | 1.0% | 401 | 0.896 |
| 60 | 40,000 | 1,000 | 2.5% | 1,000 | 0.961 |
| 60 | 40,000 | 1,000 | 5.0% | 2,074 | 0.962 |
| 60 | 40,000 | 1,000 | 10.0% | 4,086 | 0.962 |
| 60 | 40,000 | 2,000 | 1.0% | 405 | 0.779 |
| 60 | 40,000 | 2,000 | 2.5% | 1,057 | 0.963 |
| 60 | 40,000 | 2,000 | 5.0% | 2,057 | 0.987 |
| 60 | 40,000 | 2,000 | 10.0% | 4,060 | 0.989 |
| 60 | 40,000 | 4,000 | 1.0% | 432 | 0.843 |
| 60 | 40,000 | 4,000 | 2.5% | 1,053 | 0.957 |
| 60 | 40,000 | 4,000 | 5.0% | 2,033 | 0.985 |
| 60 | 40,000 | 4,000 | 10.0% | 4,003 | 0.996 |
| 60 | 80,000 | 500 | 1.0% | 804 | 0.919 |
| 60 | 80,000 | 500 | 2.5% | 2,019 | 0.919 |
| 60 | 80,000 | 1,000 | 1.0% | 807 | 0.951 |
| 60 | 80,000 | 1,000 | 2.5% | 2,038 | 0.961 |
| 60 | 80,000 | 1,000 | 5.0% | 4,099 | 0.961 |
| 60 | 80,000 | 2,000 | 1.0% | 820 | 0.938 |
| 60 | 80,000 | 2,000 | 2.5% | 2,024 | 0.981 |
| 60 | 80,000 | 2,000 | 5.0% | 4,142 | 0.984 |
| 60 | 80,000 | 2,000 | 10.0% | 8,245 | 0.984 |
| 60 | 80,000 | 4,000 | 1.0% | 822 | 0.935 |
| 60 | 80,000 | 4,000 | 2.5% | 2,067 | 0.982 |
| 60 | 80,000 | 4,000 | 5.0% | 4,012 | 0.995 |
| 60 | 80,000 | 4,000 | 10.0% | 8,039 | 0.996 |

## **NagyA1 dataset**

| **Number of sequences** | **Number of positions** | **Training size** | **Sample percentage** | **Number of positions** | **Coefficient of determination (**$\boldsymbol{r}^{\boldsymbol{2}}$**)** |
| --- | --- | --- | --- | --- | --- |
| 15 | 20,000 | 500 | 1% | 202 | 0.940 |
| 15 | 20,000 | 500 | 2.5% | 502 | 0.988 |
| 15 | 20,000 | 500 | 5% | 1,061 | 0.991 |
| 15 | 20,000 | 500 | 10% | 2,191 | 0.991 |
| 15 | 20,000 | 1,000 | 1% | 208 | 0.938 |
| 15 | 20,000 | 1,000 | 2.5% | 525 | 0.984 |
| 15 | 20,000 | 1,000 | 5% | 1,025 | 0.996 |
| 15 | 20,000 | 1,000 | 10% | 2,106 | 0.997 |
| 15 | 20,000 | 2,000 | 1% | 213 | 0.938 |
| 15 | 20,000 | 2,000 | 2.5% | 529 | 0.982 |
| 15 | 20,000 | 2,000 | 5% | 1,094 | 0.996 |
| 15 | 20,000 | 2,000 | 10% | 2,018 | 0.998 |
| 15 | 20,000 | 4,000 | 1% | 223 | 0.946 |
| 15 | 20,000 | 4,000 | 2.5% | 531 | 0.981 |
| 15 | 20,000 | 4,000 | 5% | 1,000 | 0.995 |
| 15 | 20,000 | 4,000 | 10% | 2,000 | 0.999 |
| 15 | 40,000 | 500 | 1% | 405 | 0.982 |
| 15 | 40,000 | 500 | 2.5% | 1,013 | 0.990 |
| 15 | 40,000 | 500 | 5% | 2,152 | 0.990 |
| 15 | 40,000 | 500 | 10% | 4,153 | 0.990 |
| 15 | 40,000 | 1,000 | 1% | 430 | 0.980 |
| 15 | 40,000 | 1,000 | 2.5% | 1,006 | 0.995 |
| 15 | 40,000 | 1,000 | 5% | 2,081 | 0.997 |
| 15 | 40,000 | 1,000 | 10% | 4,099 | 0.997 |
| 15 | 40,000 | 2,000 | 1% | 428 | 0.976 |
| 15 | 40,000 | 2,000 | 2.5% | 1,047 | 0.995 |
| 15 | 40,000 | 2,000 | 5% | 2,052 | 0.998 |
| 15 | 40,000 | 2,000 | 10% | 4,146 | 0.999 |
| 15 | 40,000 | 4,000 | 1% | 513 | 0.973 |
| 15 | 40,000 | 4,000 | 2.5% | 1,055 | 0.993 |
| 15 | 40,000 | 4,000 | 5% | 2,019 | 0.999 |
| 15 | 40,000 | 4,000 | 10% | 4,048 | 1.000 |
| 15 | 80,000 | 500 | 1% | 837 | 0.980 |
| 15 | 80,000 | 500 | 2.5% | 2,141 | 0.981 |
| 15 | 80,000 | 500 | 5% | 4,028 | 0.981 |
| 15 | 80,000 | 500 | 10% | 8,241 | 0.981 |
| 15 | 80,000 | 1,000 | 1% | 856 | 0.978 |
| 15 | 80,000 | 1,000 | 2.5% | 2,048 | 0.990 |
| 15 | 80,000 | 1,000 | 5% | 4,164 | 0.991 |
| 15 | 80,000 | 1,000 | 10% | 8,111 | 0.991 |
| 15 | 80,000 | 2,000 | 1% | 818 | 0.973 |
| 15 | 80,000 | 2,000 | 2.5% | 2,036 | 0.997 |
| 15 | 80,000 | 2,000 | 5% | 4,202 | 0.998 |
| 15 | 80,000 | 2,000 | 10% | 8,638 | 0.998 |
| 15 | 80,000 | 4,000 | 1% | 846 | 0.978 |
| 15 | 80,000 | 4,000 | 2.5% | 2,147 | 0.997 |
| 15 | 80,000 | 4,000 | 5% | 4,206 | 0.999 |
| 15 | 80,000 | 4,000 | 10% | 8,378 | 0.999 |
| 30 | 20,000 | 500 | 1% | 216 | 0.979 |
| 30 | 20,000 | 500 | 2.5% | 512 | 0.983 |
| 30 | 20,000 | 500 | 5% | 1,032 | 0.983 |
| 30 | 20,000 | 500 | 10% | 2,017 | 0.983 |
| 30 | 20,000 | 1,000 | 1% | 219 | 0.975 |
| 30 | 20,000 | 1,000 | 2.5% | 508 | 0.983 |
| 30 | 20,000 | 1,000 | 5% | 1,021 | 0.995 |
| 30 | 20,000 | 1,000 | 10% | 2,024 | 0.996 |
| 30 | 20,000 | 2,000 | 1% | 206 | 0.976 |
| 30 | 20,000 | 2,000 | 2.5% | 522 | 0.982 |
| 30 | 20,000 | 2,000 | 5% | 1,034 | 0.996 |
| 30 | 20,000 | 2,000 | 10% | 2,015 | 0.999 |
| 30 | 20,000 | 4,000 | 1% | 211 | 0.978 |
| 30 | 20,000 | 4,000 | 2.5% | 536 | 0.983 |
| 30 | 20,000 | 4,000 | 5% | 1,011 | 0.995 |
| 30 | 20,000 | 4,000 | 10% | 2,085 | 0.999 |
| 30 | 40,000 | 500 | 1% | 404 | 0.977 |
| 30 | 40,000 | 500 | 2.5% | 1,042 | 0.982 |
| 30 | 40,000 | 500 | 5% | 2,018 | 0.982 |
| 30 | 40,000 | 500 | 10% | 4,045 | 0.982 |
| 30 | 40,000 | 1,000 | 1% | 419 | 0.968 |
| 30 | 40,000 | 1,000 | 2.5% | 1,017 | 0.993 |
| 30 | 40,000 | 1,000 | 5% | 2,121 | 0.995 |
| 30 | 40,000 | 1,000 | 10% | 4,015 | 0.995 |
| 30 | 40,000 | 2,000 | 1% | 412 | 0.967 |
| 30 | 40,000 | 2,000 | 2.5% | 1,044 | 0.994 |
| 30 | 40,000 | 2,000 | 5% | 2,039 | 0.998 |
| 30 | 40,000 | 2,000 | 10% | 4,278 | 0.999 |
| 30 | 40,000 | 4,000 | 1% | 439 | 0.972 |
| 30 | 40,000 | 4,000 | 2.5% | 1,040 | 0.992 |
| 30 | 40,000 | 4,000 | 5% | 2,004 | 0.999 |
| 30 | 40,000 | 4,000 | 10% | 4,024 | 0.999 |
| 30 | 80,000 | 500 | 1% | 805 | 0.978 |
| 30 | 80,000 | 500 | 2.5% | 2,154 | 0.978 |
| 30 | 80,000 | 500 | 5% | 4,019 | 0.978 |
| 30 | 80,000 | 1,000 | 1% | 816 | 0.984 |
| 30 | 80,000 | 1,000 | 2.5% | 2,154 | 0.988 |
| 30 | 80,000 | 1,000 | 5% | 4,435 | 0.989 |
| 30 | 80,000 | 1,000 | 10% | 8,120 | 0.989 |
| 30 | 80,000 | 2,000 | 1% | 813 | 0.983 |
| 30 | 80,000 | 2,000 | 2.5% | 2,024 | 0.996 |
| 30 | 80,000 | 2,000 | 5% | 4,126 | 0.997 |
| 30 | 80,000 | 2,000 | 10% | 8,289 | 0.997 |
| 30 | 80,000 | 4,000 | 1% | 800 | 0.980 |
| 30 | 80,000 | 4,000 | 2.5% | 2,053 | 0.996 |
| 30 | 80,000 | 4,000 | 5% | 4,048 | 0.998 |
| 30 | 80,000 | 4,000 | 10% | 8,324 | 0.999 |
| 60 | 20,000 | 500 | 1% | 208 | 0.901 |
| 60 | 20,000 | 500 | 2.5% | 500 | 0.951 |
| 60 | 20,000 | 500 | 5% | 1,018 | 0.955 |
| 60 | 20,000 | 500 | 10% | 2,003 | 0.955 |
| 60 | 20,000 | 1,000 | 1% | 217 | 0.928 |
| 60 | 20,000 | 1,000 | 2.5% | 529 | 0.954 |
| 60 | 20,000 | 1,000 | 5% | 1,001 | 0.978 |
| 60 | 20,000 | 1,000 | 10% | 2,020 | 0.981 |
| 60 | 20,000 | 2,000 | 1% | 213 | 0.922 |
| 60 | 20,000 | 2,000 | 2.5% | 524 | 0.952 |
| 60 | 20,000 | 2,000 | 5% | 1,056 | 0.978 |
| 60 | 20,000 | 2,000 | 10% | 2,018 | 0.994 |
| 60 | 20,000 | 4,000 | 1% | 205 | 0.921 |
| 60 | 20,000 | 4,000 | 2.5% | 538 | 0.951 |
| 60 | 20,000 | 4,000 | 5% | 1,002 | 0.974 |
| 60 | 20,000 | 4,000 | 10% | 2,055 | 0.996 |
| 60 | 40,000 | 500 | 1% | 411 | 0.951 |
| 60 | 40,000 | 500 | 2.5% | 1,006 | 0.958 |
| 60 | 40,000 | 500 | 5% | 2,006 | 0.958 |
| 60 | 40,000 | 1,000 | 1% | 423 | 0.927 |
| 60 | 40,000 | 1,000 | 2.5% | 1,011 | 0.973 |
| 60 | 40,000 | 1,000 | 5% | 2,019 | 0.976 |
| 60 | 40,000 | 1,000 | 10% | 4,016 | 0.976 |
| 60 | 40,000 | 2,000 | 1% | 414 | 0.926 |
| 60 | 40,000 | 2,000 | 2.5% | 1,027 | 0.971 |
| 60 | 40,000 | 2,000 | 5% | 2,001 | 0.990 |
| 60 | 40,000 | 2,000 | 10% | 4,097 | 0.993 |
| 60 | 40,000 | 4,000 | 1% | 401 | 0.925 |
| 60 | 40,000 | 4,000 | 2.5% | 1,033 | 0.976 |
| 60 | 40,000 | 4,000 | 5% | 2,014 | 0.993 |
| 60 | 40,000 | 4,000 | 10% | 4,102 | 0.999 |
| 60 | 80,000 | 500 | 1% | 839 | 0.949 |
| 60 | 80,000 | 500 | 2.5% | 2,008 | 0.949 |
| 60 | 80,000 | 1,000 | 1% | 804 | 0.966 |
| 60 | 80,000 | 1,000 | 2.5% | 2,080 | 0.973 |
| 60 | 80,000 | 1,000 | 5% | 4,061 | 0.973 |
| 60 | 80,000 | 2,000 | 1% | 820 | 0.957 |
| 60 | 80,000 | 2,000 | 2.5% | 2,005 | 0.986 |
| 60 | 80,000 | 2,000 | 5% | 4,289 | 0.988 |
| 60 | 80,000 | 2,000 | 10% | 8,310 | 0.988 |
| 60 | 80,000 | 4,000 | 1% | 821 | 0.959 |
| 60 | 80,000 | 4,000 | 2.5% | 2,047 | 0.989 |
| 60 | 80,000 | 4,000 | 5% | 4,047 | 0.998 |
| 60 | 80,000 | 4,000 | 10% | 8,146 | 0.998 |
|  |  |  |  |  |  |

## **ShenA9 dataset**

| **Number of sequences** | **Number of positions** | **Training size** | **Sample percentage** | **Number of positions** | **Coefficient of determination (**$\boldsymbol{r}^{\boldsymbol{2}}$**)** |
| --- | --- | --- | --- | --- | --- |
| 15 | 20,000 | 500 | 1% | 210 | 0.948 |
| 15 | 20,000 | 500 | 2.5% | 510 | 0.981 |
| 15 | 20,000 | 500 | 5% | 1,062 | 0.984 |
| 15 | 20,000 | 500 | 10% | 2,018 | 0.984 |
| 15 | 20,000 | 1,000 | 1% | 203 | 0.934 |
| 15 | 20,000 | 1,000 | 2.5% | 552 | 0.980 |
| 15 | 20,000 | 1,000 | 5% | 1,025 | 0.994 |
| 15 | 20,000 | 1,000 | 10% | 2,076 | 0.996 |
| 15 | 20,000 | 2,000 | 1% | 201 | 0.914 |
| 15 | 20,000 | 2,000 | 2.5% | 530 | 0.975 |
| 15 | 20,000 | 2,000 | 5% | 1,076 | 0.994 |
| 15 | 20,000 | 2,000 | 10% | 2,012 | 0.998 |
| 15 | 20,000 | 4,000 | 1% | 201 | 0.921 |
| 15 | 20,000 | 4,000 | 2.5% | 533 | 0.971 |
| 15 | 20,000 | 4,000 | 5% | 1,068 | 0.994 |
| 15 | 20,000 | 4,000 | 10% | 2,041 | 0.998 |
| 15 | 40,000 | 500 | 1% | 405 | 0.969 |
| 15 | 40,000 | 500 | 2.5% | 1,045 | 0.980 |
| 15 | 40,000 | 500 | 5% | 2,090 | 0.981 |
| 15 | 40,000 | 500 | 10% | 4,021 | 0.981 |
| 15 | 40,000 | 1,000 | 1% | 424 | 0.963 |
| 15 | 40,000 | 1,000 | 2.5% | 1,006 | 0.991 |
| 15 | 40,000 | 1,000 | 5% | 2,027 | 0.994 |
| 15 | 40,000 | 1,000 | 10% | 4,176 | 0.994 |
| 15 | 40,000 | 2,000 | 1% | 430 | 0.956 |
| 15 | 40,000 | 2,000 | 2.5% | 1,027 | 0.991 |
| 15 | 40,000 | 2,000 | 5% | 2,054 | 0.997 |
| 15 | 40,000 | 2,000 | 10% | 4,165 | 0.997 |
| 15 | 40,000 | 4,000 | 1% | 436 | 0.944 |
| 15 | 40,000 | 4,000 | 2.5% | 1,016 | 0.988 |
| 15 | 40,000 | 4,000 | 5% | 2,062 | 0.997 |
| 15 | 40,000 | 4,000 | 10% | 4,174 | 0.999 |
| 15 | 80,000 | 500 | 1% | 819 | 0.979 |
| 15 | 80,000 | 500 | 2.5% | 2,091 | 0.980 |
| 15 | 80,000 | 500 | 5% | 4,089 | 0.981 |
| 15 | 80,000 | 500 | 10% | 8,173 | 0.981 |
| 15 | 80,000 | 1,000 | 1% | 827 | 0.982 |
| 15 | 80,000 | 1,000 | 2.5% | 2,079 | 0.993 |
| 15 | 80,000 | 1,000 | 5% | 4,185 | 0.993 |
| 15 | 80,000 | 1,000 | 10% | 8,621 | 0.994 |
| 15 | 80,000 | 2,000 | 1% | 806 | 0.978 |
| 15 | 80,000 | 2,000 | 2.5% | 2,015 | 0.996 |
| 15 | 80,000 | 2,000 | 5% | 4,363 | 0.998 |
| 15 | 80,000 | 2,000 | 10% | 8,295 | 0.998 |
| 15 | 80,000 | 4,000 | 1% | 831 | 0.976 |
| 15 | 80,000 | 4,000 | 2.5% | 2,019 | 0.997 |
| 15 | 80,000 | 4,000 | 5% | 4,030 | 0.999 |
| 15 | 80,000 | 4,000 | 10% | 8,245 | 0.999 |
| 30 | 20,000 | 500 | 1% | 219 | 0.967 |
| 30 | 20,000 | 500 | 2.5% | 506 | 0.981 |
| 30 | 20,000 | 500 | 5% | 1,030 | 0.982 |
| 30 | 20,000 | 500 | 10% | 2,103 | 0.982 |
| 30 | 20,000 | 1,000 | 1% | 201 | 0.955 |
| 30 | 20,000 | 1,000 | 2.5% | 527 | 0.983 |
| 30 | 20,000 | 1,000 | 5% | 1,003 | 0.993 |
| 30 | 20,000 | 1,000 | 10% | 2,080 | 0.995 |
| 30 | 20,000 | 2,000 | 1% | 209 | 0.968 |
| 30 | 20,000 | 2,000 | 2.5% | 505 | 0.973 |
| 30 | 20,000 | 2,000 | 5% | 1,068 | 0.988 |
| 30 | 20,000 | 2,000 | 10% | 2,072 | 0.997 |
| 30 | 20,000 | 4,000 | 1% | 207 | 0.965 |
| 30 | 20,000 | 4,000 | 2.5% | 501 | 0.967 |
| 30 | 20,000 | 4,000 | 5% | 1,055 | 0.985 |
| 30 | 20,000 | 4,000 | 10% | 2,093 | 0.997 |
| 30 | 40,000 | 500 | 1% | 401 | 0.978 |
| 30 | 40,000 | 500 | 2.5% | 1,007 | 0.981 |
| 30 | 40,000 | 500 | 5% | 2,137 | 0.981 |
| 30 | 40,000 | 500 | 10% | 4,102 | 0.981 |
| 30 | 40,000 | 1,000 | 1% | 413 | 0.973 |
| 30 | 40,000 | 1,000 | 2.5% | 1,011 | 0.989 |
| 30 | 40,000 | 1,000 | 5% | 2,094 | 0.991 |
| 30 | 40,000 | 1,000 | 10% | 4,259 | 0.991 |
| 30 | 40,000 | 2,000 | 1% | 407 | 0.966 |
| 30 | 40,000 | 2,000 | 2.5% | 1,036 | 0.988 |
| 30 | 40,000 | 2,000 | 5% | 2,044 | 0.997 |
| 30 | 40,000 | 2,000 | 10% | 4,149 | 0.998 |
| 30 | 40,000 | 4,000 | 1% | 427 | 0.961 |
| 30 | 40,000 | 4,000 | 2.5% | 1,008 | 0.983 |
| 30 | 40,000 | 4,000 | 5% | 2,049 | 0.996 |
| 30 | 40,000 | 4,000 | 10% | 4,079 | 0.999 |
| 30 | 80,000 | 500 | 1% | 849 | 0.982 |
| 30 | 80,000 | 500 | 2.5% | 2,159 | 0.982 |
| 30 | 80,000 | 500 | 5% | 4,003 | 0.982 |
| 30 | 80,000 | 1,000 | 1% | 828 | 0.987 |
| 30 | 80,000 | 1,000 | 2.5% | 2,003 | 0.992 |
| 30 | 80,000 | 1,000 | 5% | 4,111 | 0.993 |
| 30 | 80,000 | 1,000 | 10% | 8,138 | 0.993 |
| 30 | 80,000 | 2,000 | 1% | 848 | 0.981 |
| 30 | 80,000 | 2,000 | 2.5% | 2,049 | 0.997 |
| 30 | 80,000 | 2,000 | 5% | 4,012 | 0.997 |
| 30 | 80,000 | 2,000 | 10% | 8,054 | 0.997 |
| 30 | 80,000 | 4,000 | 1% | 841 | 0.975 |
| 30 | 80,000 | 4,000 | 2.5% | 2,039 | 0.996 |
| 30 | 80,000 | 4,000 | 5% | 4,107 | 0.999 |
| 30 | 80,000 | 4,000 | 10% | 8,276 | 0.999 |
| 60 | 20,000 | 500 | 1% | 217 | 0.881 |
| 60 | 20,000 | 500 | 2.5% | 503 | 0.972 |
| 60 | 20,000 | 500 | 5% | 1,004 | 0.973 |
| 60 | 20,000 | 500 | 10% | 2,002 | 0.973 |
| 60 | 20,000 | 1,000 | 1% | 221 | 0.906 |
| 60 | 20,000 | 1,000 | 2.5% | 506 | 0.977 |
| 60 | 20,000 | 1,000 | 5% | 1,008 | 0.987 |
| 60 | 20,000 | 1,000 | 10% | 2,025 | 0.988 |
| 60 | 20,000 | 2,000 | 1% | 226 | 0.905 |
| 60 | 20,000 | 2,000 | 2.5% | 520 | 0.973 |
| 60 | 20,000 | 2,000 | 5% | 1,005 | 0.983 |
| 60 | 20,000 | 2,000 | 10% | 2,016 | 0.994 |
| 60 | 20,000 | 4,000 | 1% | 209 | 0.883 |
| 60 | 20,000 | 4,000 | 2.5% | 520 | 0.973 |
| 60 | 20,000 | 4,000 | 5% | 1,022 | 0.980 |
| 60 | 20,000 | 4,000 | 10% | 2,026 | 0.993 |
| 60 | 40,000 | 500 | 1% | 413 | 0.969 |
| 60 | 40,000 | 500 | 2.5% | 1,001 | 0.971 |
| 60 | 40,000 | 500 | 5% | 2,002 | 0.971 |
| 60 | 40,000 | 1,000 | 1% | 430 | 0.971 |
| 60 | 40,000 | 1,000 | 2.5% | 1,021 | 0.987 |
| 60 | 40,000 | 1,000 | 5% | 2,087 | 0.988 |
| 60 | 40,000 | 1,000 | 10% | 4,069 | 0.988 |
| 60 | 40,000 | 2,000 | 1% | 430 | 0.968 |
| 60 | 40,000 | 2,000 | 2.5% | 1,028 | 0.983 |
| 60 | 40,000 | 2,000 | 5% | 2,021 | 0.992 |
| 60 | 40,000 | 2,000 | 10% | 4,172 | 0.993 |
| 60 | 40,000 | 4,000 | 1% | 438 | 0.969 |
| 60 | 40,000 | 4,000 | 2.5% | 1,054 | 0.982 |
| 60 | 40,000 | 4,000 | 5% | 2,085 | 0.991 |
| 60 | 40,000 | 4,000 | 10% | 4,010 | 0.998 |
| 60 | 80,000 | 500 | 1% | 806 | 0.966 |
| 60 | 80,000 | 500 | 2.5% | 2,009 | 0.967 |
| 60 | 80,000 | 1,000 | 1% | 810 | 0.982 |
| 60 | 80,000 | 1,000 | 2.5% | 2,038 | 0.985 |
| 60 | 80,000 | 1,000 | 5% | 4,106 | 0.985 |
| 60 | 80,000 | 2,000 | 1% | 856 | 0.977 |
| 60 | 80,000 | 2,000 | 2.5% | 2,010 | 0.992 |
| 60 | 80,000 | 2,000 | 5% | 4,005 | 0.994 |
| 60 | 80,000 | 2,000 | 10% | 8,356 | 0.994 |
| 60 | 80,000 | 4,000 | 1% | 811 | 0.974 |
| 60 | 80,000 | 4,000 | 2.5% | 2,091 | 0.990 |
| 60 | 80,000 | 4,000 | 5% | 4,077 | 0.996 |
| 60 | 80,000 | 4,000 | 10% | 8,370 | 0.996 |

## **StruA5 dataset**

| **Number of sequences** | **Number of positions** | **Training size** | **Sample percentage** | **Number of positions** | **Coefficient of determination (**$\boldsymbol{r}^{\boldsymbol{2}}$**)** |
| --- | --- | --- | --- | --- | --- |
| 15 | 20,000 | 500 | 1% | 213 | 0.924 |
| 15 | 20,000 | 500 | 2.5% | 500 | 0.966 |
| 15 | 20,000 | 500 | 5% | 1,057 | 0.973 |
| 15 | 20,000 | 500 | 10% | 2,061 | 0.974 |
| 15 | 20,000 | 1,000 | 1% | 213 | 0.911 |
| 15 | 20,000 | 1,000 | 2.5% | 516 | 0.964 |
| 15 | 20,000 | 1,000 | 5% | 1,028 | 0.983 |
| 15 | 20,000 | 1,000 | 10% | 2,089 | 0.988 |
| 15 | 20,000 | 2,000 | 1% | 201 | 0.903 |
| 15 | 20,000 | 2,000 | 2.5% | 532 | 0.959 |
| 15 | 20,000 | 2,000 | 5% | 1,041 | 0.983 |
| 15 | 20,000 | 2,000 | 10% | 2,067 | 0.994 |
| 15 | 20,000 | 4,000 | 1% | 216 | 0.899 |
| 15 | 20,000 | 4,000 | 2.5% | 502 | 0.952 |
| 15 | 20,000 | 4,000 | 5% | 1,006 | 0.983 |
| 15 | 20,000 | 4,000 | 10% | 2,107 | 0.995 |
| 15 | 40,000 | 500 | 1% | 401 | 0.915 |
| 15 | 40,000 | 500 | 2.5% | 1,029 | 0.947 |
| 15 | 40,000 | 500 | 5% | 2,084 | 0.949 |
| 15 | 40,000 | 500 | 10% | 4,156 | 0.949 |
| 15 | 40,000 | 1,000 | 1% | 412 | 0.905 |
| 15 | 40,000 | 1,000 | 2.5% | 1,028 | 0.958 |
| 15 | 40,000 | 1,000 | 5% | 2,109 | 0.972 |
| 15 | 40,000 | 1,000 | 10% | 4,195 | 0.974 |
| 15 | 40,000 | 2,000 | 1% | 414 | 0.876 |
| 15 | 40,000 | 2,000 | 2.5% | 1,070 | 0.971 |
| 15 | 40,000 | 2,000 | 5% | 2,060 | 0.989 |
| 15 | 40,000 | 2,000 | 10% | 4,106 | 0.991 |
| 15 | 40,000 | 4,000 | 1% | 424 | 0.870 |
| 15 | 40,000 | 4,000 | 2.5% | 1,004 | 0.962 |
| 15 | 40,000 | 4,000 | 5% | 2,032 | 0.990 |
| 15 | 40,000 | 4,000 | 10% | 4,050 | 0.997 |
| 15 | 80,000 | 500 | 1% | 804 | 0.925 |
| 15 | 80,000 | 500 | 2.5% | 2,094 | 0.931 |
| 15 | 80,000 | 500 | 5% | 4,042 | 0.932 |
| 15 | 80,000 | 500 | 10% | 8,311 | 0.933 |
| 15 | 80,000 | 1,000 | 1% | 815 | 0.927 |
| 15 | 80,000 | 1,000 | 2.5% | 2,042 | 0.970 |
| 15 | 80,000 | 1,000 | 5% | 4,201 | 0.972 |
| 15 | 80,000 | 1,000 | 10% | 8,096 | 0.972 |
| 15 | 80,000 | 2,000 | 1% | 810 | 0.909 |
| 15 | 80,000 | 2,000 | 2.5% | 2,003 | 0.980 |
| 15 | 80,000 | 2,000 | 5% | 4,021 | 0.989 |
| 15 | 80,000 | 2,000 | 10% | 8,938 | 0.991 |
| 15 | 80,000 | 4,000 | 1% | 829 | 0.915 |
| 15 | 80,000 | 4,000 | 2.5% | 2,111 | 0.985 |
| 15 | 80,000 | 4,000 | 5% | 4,179 | 0.995 |
| 15 | 80,000 | 4,000 | 10% | 8,490 | 0.997 |
| 30 | 20,000 | 500 | 1% | 201 | 0.860 |
| 30 | 20,000 | 500 | 2.5% | 501 | 0.937 |
| 30 | 20,000 | 500 | 5% | 1,001 | 0.941 |
| 30 | 20,000 | 500 | 10% | 2,019 | 0.942 |
| 30 | 20,000 | 1,000 | 1% | 211 | 0.853 |
| 30 | 20,000 | 1,000 | 2.5% | 503 | 0.946 |
| 30 | 20,000 | 1,000 | 5% | 1,017 | 0.972 |
| 30 | 20,000 | 1,000 | 10% | 2,102 | 0.975 |
| 30 | 20,000 | 2,000 | 1% | 216 | 0.838 |
| 30 | 20,000 | 2,000 | 2.5% | 522 | 0.921 |
| 30 | 20,000 | 2,000 | 5% | 1,018 | 0.965 |
| 30 | 20,000 | 2,000 | 10% | 2,051 | 0.989 |
| 30 | 20,000 | 4,000 | 1% | 204 | 0.834 |
| 30 | 20,000 | 4,000 | 2.5% | 534 | 0.916 |
| 30 | 20,000 | 4,000 | 5% | 1,001 | 0.968 |
| 30 | 20,000 | 4,000 | 10% | 2,014 | 0.991 |
| 30 | 40,000 | 500 | 1% | 406 | 0.890 |
| 30 | 40,000 | 500 | 2.5% | 1,056 | 0.919 |
| 30 | 40,000 | 500 | 5% | 2,047 | 0.919 |
| 30 | 40,000 | 500 | 10% | 4,055 | 0.919 |
| 30 | 40,000 | 1,000 | 1% | 419 | 0.912 |
| 30 | 40,000 | 1,000 | 2.5% | 1,027 | 0.970 |
| 30 | 40,000 | 1,000 | 5% | 2,191 | 0.973 |
| 30 | 40,000 | 1,000 | 10% | 4,041 | 0.973 |
| 30 | 40,000 | 2,000 | 1% | 424 | 0.883 |
| 30 | 40,000 | 2,000 | 2.5% | 1,034 | 0.967 |
| 30 | 40,000 | 2,000 | 5% | 2,076 | 0.987 |
| 30 | 40,000 | 2,000 | 10% | 4,109 | 0.989 |
| 30 | 40,000 | 4,000 | 1% | 402 | 0.890 |
| 30 | 40,000 | 4,000 | 2.5% | 1,023 | 0.968 |
| 30 | 40,000 | 4,000 | 5% | 2,019 | 0.989 |
| 30 | 40,000 | 4,000 | 10% | 4,068 | 0.996 |
| 30 | 80,000 | 500 | 1% | 809 | 0.869 |
| 30 | 80,000 | 500 | 2.5% | 2,063 | 0.870 |
| 30 | 80,000 | 500 | 5% | 4,002 | 0.871 |
| 30 | 80,000 | 1,000 | 1% | 840 | 0.940 |
| 30 | 80,000 | 1,000 | 2.5% | 2,060 | 0.950 |
| 30 | 80,000 | 1,000 | 5% | 4,110 | 0.951 |
| 30 | 80,000 | 1,000 | 10% | 8,127 | 0.951 |
| 30 | 80,000 | 2,000 | 1% | 801 | 0.924 |
| 30 | 80,000 | 2,000 | 2.5% | 2,082 | 0.983 |
| 30 | 80,000 | 2,000 | 5% | 4,137 | 0.986 |
| 30 | 80,000 | 2,000 | 10% | 8,037 | 0.986 |
| 30 | 80,000 | 4,000 | 1% | 861 | 0.924 |
| 30 | 80,000 | 4,000 | 2.5% | 2,067 | 0.979 |
| 30 | 80,000 | 4,000 | 5% | 4,103 | 0.990 |
| 30 | 80,000 | 4,000 | 10% | 8,042 | 0.992 |
| 60 | 20,000 | 500 | 1% | 202 | 0.780 |
| 60 | 20,000 | 500 | 2.5% | 509 | 0.897 |
| 60 | 20,000 | 500 | 5% | 1,051 | 0.899 |
| 60 | 20,000 | 500 | 10% | 2,002 | 0.900 |
| 60 | 20,000 | 1,000 | 1% | 227 | 0.801 |
| 60 | 20,000 | 1,000 | 2.5% | 500 | 0.880 |
| 60 | 20,000 | 1,000 | 5% | 1,014 | 0.931 |
| 60 | 20,000 | 1,000 | 10% | 2,118 | 0.938 |
| 60 | 20,000 | 2,000 | 1% | 205 | 0.784 |
| 60 | 20,000 | 2,000 | 2.5% | 547 | 0.886 |
| 60 | 20,000 | 2,000 | 5% | 1,002 | 0.931 |
| 60 | 20,000 | 2,000 | 10% | 2,044 | 0.979 |
| 60 | 20,000 | 4,000 | 1% | 218 | 0.806 |
| 60 | 20,000 | 4,000 | 2.5% | 553 | 0.875 |
| 60 | 20,000 | 4,000 | 5% | 1,071 | 0.923 |
| 60 | 20,000 | 4,000 | 10% | 2,041 | 0.973 |
| 60 | 40,000 | 500 | 1% | 419 | 0.858 |
| 60 | 40,000 | 500 | 2.5% | 1,041 | 0.885 |
| 60 | 40,000 | 1,000 | 1% | 429 | 0.842 |
| 60 | 40,000 | 1,000 | 2.5% | 1,000 | 0.937 |
| 60 | 40,000 | 1,000 | 5% | 2,016 | 0.942 |
| 60 | 40,000 | 1,000 | 10% | 4,028 | 0.942 |
| 60 | 40,000 | 2,000 | 1% | 406 | 0.830 |
| 60 | 40,000 | 2,000 | 2.5% | 1,004 | 0.925 |
| 60 | 40,000 | 2,000 | 5% | 2,009 | 0.970 |
| 60 | 40,000 | 2,000 | 10% | 4,170 | 0.974 |
| 60 | 40,000 | 4,000 | 1% | 434 | 0.832 |
| 60 | 40,000 | 4,000 | 2.5% | 1,075 | 0.916 |
| 60 | 40,000 | 4,000 | 5% | 2,017 | 0.967 |
| 60 | 40,000 | 4,000 | 10% | 4,067 | 0.990 |
| 60 | 80,000 | 500 | 1% | 829 | 0.858 |
| 60 | 80,000 | 500 | 2.5% | 2,001 | 0.858 |
| 60 | 80,000 | 1,000 | 1% | 813 | 0.881 |
| 60 | 80,000 | 1,000 | 2.5% | 2,041 | 0.901 |
| 60 | 80,000 | 1,000 | 5% | 4,057 | 0.901 |
| 60 | 80,000 | 2,000 | 1% | 864 | 0.882 |
| 60 | 80,000 | 2,000 | 2.5% | 2,004 | 0.959 |
| 60 | 80,000 | 2,000 | 5% | 4,154 | 0.963 |
| 60 | 80,000 | 2,000 | 10% | 8,046 | 0.964 |
| 60 | 80,000 | 4,000 | 1% | 827 | 0.877 |
| 60 | 80,000 | 4,000 | 2.5% | 2,064 | 0.957 |
| 60 | 80,000 | 4,000 | 5% | 4,019 | 0.984 |
| 60 | 80,000 | 4,000 | 10% | 8,182 | 0.986 |

## **WickA3 dataset**

| **Number of sequences** | **Number of positions** | **Training size** | **Sample percentage** | **Number of positions** | **Coefficient of determination (**$\boldsymbol{r}^{\boldsymbol{2}}$**)** |
| --- | --- | --- | --- | --- | --- |
| 15 | 20,000 | 500 | 1% | 204 | 0.933 |
| 15 | 20,000 | 500 | 2.5% | 500 | 0.983 |
| 15 | 20,000 | 500 | 5% | 1,068 | 0.988 |
| 15 | 20,000 | 500 | 10% | 2,132 | 0.988 |
| 15 | 20,000 | 1,000 | 1% | 201 | 0.927 |
| 15 | 20,000 | 1,000 | 2.5% | 501 | 0.977 |
| 15 | 20,000 | 1,000 | 5% | 1,030 | 0.994 |
| 15 | 20,000 | 1,000 | 10% | 2,100 | 0.995 |
| 15 | 20,000 | 2,000 | 1% | 210 | 0.919 |
| 15 | 20,000 | 2,000 | 2.5% | 554 | 0.973 |
| 15 | 20,000 | 2,000 | 5% | 1,038 | 0.992 |
| 15 | 20,000 | 2,000 | 10% | 2,085 | 0.998 |
| 15 | 20,000 | 4,000 | 1% | 217 | 0.916 |
| 15 | 20,000 | 4,000 | 2.5% | 531 | 0.969 |
| 15 | 20,000 | 4,000 | 5% | 1,017 | 0.990 |
| 15 | 20,000 | 4,000 | 10% | 2,009 | 0.998 |
| 15 | 40,000 | 500 | 1% | 422 | 0.970 |
| 15 | 40,000 | 500 | 2.5% | 1,003 | 0.982 |
| 15 | 40,000 | 500 | 5% | 2,128 | 0.982 |
| 15 | 40,000 | 500 | 10% | 4,114 | 0.983 |
| 15 | 40,000 | 1,000 | 1% | 422 | 0.949 |
| 15 | 40,000 | 1,000 | 2.5% | 1,043 | 0.992 |
| 15 | 40,000 | 1,000 | 5% | 2,053 | 0.994 |
| 15 | 40,000 | 1,000 | 10% | 4,095 | 0.995 |
| 15 | 40,000 | 2,000 | 1% | 410 | 0.935 |
| 15 | 40,000 | 2,000 | 2.5% | 1,052 | 0.992 |
| 15 | 40,000 | 2,000 | 5% | 2,061 | 0.998 |
| 15 | 40,000 | 2,000 | 10% | 4,176 | 0.999 |
| 15 | 40,000 | 4,000 | 1% | 425 | 0.934 |
| 15 | 40,000 | 4,000 | 2.5% | 1,064 | 0.990 |
| 15 | 40,000 | 4,000 | 5% | 2,035 | 0.998 |
| 15 | 40,000 | 4,000 | 10% | 4,175 | 1.000 |
| 15 | 80,000 | 500 | 1% | 826 | 0.975 |
| 15 | 80,000 | 500 | 2.5% | 2,149 | 0.976 |
| 15 | 80,000 | 500 | 5% | 4,066 | 0.976 |
| 15 | 80,000 | 500 | 10% | 8,046 | 0.977 |
| 15 | 80,000 | 1,000 | 1% | 808 | 0.979 |
| 15 | 80,000 | 1,000 | 2.5% | 2,146 | 0.991 |
| 15 | 80,000 | 1,000 | 5% | 4,150 | 0.991 |
| 15 | 80,000 | 1,000 | 10% | 8,555 | 0.991 |
| 15 | 80,000 | 2,000 | 1% | 821 | 0.977 |
| 15 | 80,000 | 2,000 | 2.5% | 2,029 | 0.997 |
| 15 | 80,000 | 2,000 | 5% | 4,118 | 0.998 |
| 15 | 80,000 | 2,000 | 10% | 8,120 | 0.998 |
| 15 | 80,000 | 4,000 | 1% | 803 | 0.968 |
| 15 | 80,000 | 4,000 | 2.5% | 2,038 | 0.997 |
| 15 | 80,000 | 4,000 | 5% | 4,165 | 0.999 |
| 15 | 80,000 | 4,000 | 10% | 8,078 | 1.000 |
| 30 | 20,000 | 500 | 1% | 208 | 0.956 |
| 30 | 20,000 | 500 | 2.5% | 504 | 0.977 |
| 30 | 20,000 | 500 | 5% | 1,074 | 0.980 |
| 30 | 20,000 | 500 | 10% | 2,052 | 0.980 |
| 30 | 20,000 | 1,000 | 1% | 215 | 0.932 |
| 30 | 20,000 | 1,000 | 2.5% | 507 | 0.969 |
| 30 | 20,000 | 1,000 | 5% | 1,031 | 0.990 |
| 30 | 20,000 | 1,000 | 10% | 2,129 | 0.991 |
| 30 | 20,000 | 2,000 | 1% | 211 | 0.924 |
| 30 | 20,000 | 2,000 | 2.5% | 522 | 0.960 |
| 30 | 20,000 | 2,000 | 5% | 1,045 | 0.988 |
| 30 | 20,000 | 2,000 | 10% | 2,015 | 0.997 |
| 30 | 20,000 | 4,000 | 1% | 236 | 0.924 |
| 30 | 20,000 | 4,000 | 2.5% | 509 | 0.953 |
| 30 | 20,000 | 4,000 | 5% | 1,063 | 0.986 |
| 30 | 20,000 | 4,000 | 10% | 2,129 | 0.997 |
| 30 | 40,000 | 500 | 1% | 403 | 0.977 |
| 30 | 40,000 | 500 | 2.5% | 1,009 | 0.979 |
| 30 | 40,000 | 500 | 5% | 2,062 | 0.979 |
| 30 | 40,000 | 1,000 | 1% | 428 | 0.959 |
| 30 | 40,000 | 1,000 | 2.5% | 1,022 | 0.990 |
| 30 | 40,000 | 1,000 | 5% | 2,100 | 0.992 |
| 30 | 40,000 | 1,000 | 10% | 4,082 | 0.992 |
| 30 | 40,000 | 2,000 | 1% | 411 | 0.952 |
| 30 | 40,000 | 2,000 | 2.5% | 1,053 | 0.987 |
| 30 | 40,000 | 2,000 | 5% | 2,034 | 0.996 |
| 30 | 40,000 | 2,000 | 10% | 4,091 | 0.997 |
| 30 | 40,000 | 4,000 | 1% | 406 | 0.951 |
| 30 | 40,000 | 4,000 | 2.5% | 1,040 | 0.986 |
| 30 | 40,000 | 4,000 | 5% | 2,039 | 0.996 |
| 30 | 40,000 | 4,000 | 10% | 4,072 | 0.999 |
| 30 | 80,000 | 500 | 1% | 861 | 0.963 |
| 30 | 80,000 | 500 | 2.5% | 2,008 | 0.963 |
| 30 | 80,000 | 1,000 | 1% | 814 | 0.981 |
| 30 | 80,000 | 1,000 | 2.5% | 2,091 | 0.988 |
| 30 | 80,000 | 1,000 | 5% | 4,222 | 0.988 |
| 30 | 80,000 | 1,000 | 10% | 8,121 | 0.988 |
| 30 | 80,000 | 2,000 | 1% | 832 | 0.978 |
| 30 | 80,000 | 2,000 | 2.5% | 2,000 | 0.995 |
| 30 | 80,000 | 2,000 | 5% | 4,107 | 0.996 |
| 30 | 80,000 | 2,000 | 10% | 8,507 | 0.996 |
| 30 | 80,000 | 4,000 | 1% | 857 | 0.972 |
| 30 | 80,000 | 4,000 | 2.5% | 2,114 | 0.994 |
| 30 | 80,000 | 4,000 | 5% | 4,029 | 0.999 |
| 30 | 80,000 | 4,000 | 10% | 8,435 | 0.999 |
| 60 | 20,000 | 500 | 1% | 206 | 0.926 |
| 60 | 20,000 | 500 | 2.5% | 509 | 0.953 |
| 60 | 20,000 | 500 | 5% | 1,028 | 0.954 |
| 60 | 20,000 | 1,000 | 1% | 214 | 0.916 |
| 60 | 20,000 | 1,000 | 2.5% | 532 | 0.943 |
| 60 | 20,000 | 1,000 | 5% | 1,008 | 0.970 |
| 60 | 20,000 | 1,000 | 10% | 2,067 | 0.973 |
| 60 | 20,000 | 2,000 | 1% | 218 | 0.915 |
| 60 | 20,000 | 2,000 | 2.5% | 554 | 0.950 |
| 60 | 20,000 | 2,000 | 5% | 1,054 | 0.975 |
| 60 | 20,000 | 2,000 | 10% | 2,039 | 0.992 |
| 60 | 20,000 | 4,000 | 1% | 204 | 0.909 |
| 60 | 20,000 | 4,000 | 2.5% | 507 | 0.943 |
| 60 | 20,000 | 4,000 | 5% | 1,040 | 0.966 |
| 60 | 20,000 | 4,000 | 10% | 2,084 | 0.994 |
| 60 | 40,000 | 500 | 1% | 402 | 0.947 |
| 60 | 40,000 | 500 | 2.5% | 1,043 | 0.955 |
| 60 | 40,000 | 1,000 | 1% | 423 | 0.949 |
| 60 | 40,000 | 1,000 | 2.5% | 1,010 | 0.973 |
| 60 | 40,000 | 1,000 | 5% | 2,036 | 0.975 |
| 60 | 40,000 | 1,000 | 10% | 4,009 | 0.975 |
| 60 | 40,000 | 2,000 | 1% | 416 | 0.943 |
| 60 | 40,000 | 2,000 | 2.5% | 1,074 | 0.968 |
| 60 | 40,000 | 2,000 | 5% | 2,019 | 0.988 |
| 60 | 40,000 | 2,000 | 10% | 4,166 | 0.990 |
| 60 | 40,000 | 4,000 | 1% | 446 | 0.943 |
| 60 | 40,000 | 4,000 | 2.5% | 1,027 | 0.957 |
| 60 | 40,000 | 4,000 | 5% | 2,063 | 0.984 |
| 60 | 40,000 | 4,000 | 10% | 4,051 | 0.997 |
| 60 | 80,000 | 500 | 1% | 806 | 0.926 |
| 60 | 80,000 | 500 | 2.5% | 2,002 | 0.926 |
| 60 | 80,000 | 1,000 | 1% | 803 | 0.954 |
| 60 | 80,000 | 1,000 | 2.5% | 2,020 | 0.962 |
| 60 | 80,000 | 1,000 | 5% | 4,075 | 0.962 |
| 60 | 80,000 | 2,000 | 1% | 811 | 0.952 |
| 60 | 80,000 | 2,000 | 2.5% | 2,013 | 0.986 |
| 60 | 80,000 | 2,000 | 5% | 4,223 | 0.988 |
| 60 | 80,000 | 2,000 | 10% | 8,119 | 0.988 |
| 60 | 80,000 | 4,000 | 1% | 880 | 0.939 |
| 60 | 80,000 | 4,000 | 2.5% | 2,109 | 0.984 |
| 60 | 80,000 | 4,000 | 5% | 4,013 | 0.996 |
| 60 | 80,000 | 4,000 | 10% | 8,292 | 0.996 |

## **YangA8 dataset**

| Number of sequences | Number of positions | Training size | Sample percentage | Number of positions | Coefficient of determination ($r^{2}$) |
| --- | --- | --- | --- | --- | --- |
| 15 | 20,000 | 500 | 1% | 212 | 0.919 |
| 15 | 20,000 | 500 | 2.5% | 505 | 0.961 |
| 15 | 20,000 | 500 | 5% | 1,040 | 0.969 |
| 15 | 20,000 | 500 | 10% | 2,133 | 0.970 |
| 15 | 20,000 | 1,000 | 1% | 211 | 0.925 |
| 15 | 20,000 | 1,000 | 2.5% | 512 | 0.966 |
| 15 | 20,000 | 1,000 | 5% | 1,014 | 0.988 |
| 15 | 20,000 | 1,000 | 10% | 2,008 | 0.992 |
| 15 | 20,000 | 2,000 | 1% | 221 | 0.925 |
| 15 | 20,000 | 2,000 | 2.5% | 551 | 0.963 |
| 15 | 20,000 | 2,000 | 5% | 1,050 | 0.989 |
| 15 | 20,000 | 2,000 | 10% | 2,064 | 0.998 |
| 15 | 20,000 | 4,000 | 1% | 210 | 0.917 |
| 15 | 20,000 | 4,000 | 2.5% | 513 | 0.966 |
| 15 | 20,000 | 4,000 | 5% | 1,019 | 0.987 |
| 15 | 20,000 | 4,000 | 10% | 2,099 | 0.998 |
| 15 | 40,000 | 500 | 1% | 402 | 0.954 |
| 15 | 40,000 | 500 | 2.5% | 1,127 | 0.967 |
| 15 | 40,000 | 500 | 5% | 2,012 | 0.968 |
| 15 | 40,000 | 500 | 10% | 4,221 | 0.969 |
| 15 | 40,000 | 1,000 | 1% | 422 | 0.948 |
| 15 | 40,000 | 1,000 | 2.5% | 1,014 | 0.985 |
| 15 | 40,000 | 1,000 | 5% | 2,163 | 0.991 |
| 15 | 40,000 | 1,000 | 10% | 4,020 | 0.991 |
| 15 | 40,000 | 2,000 | 1% | 400 | 0.944 |
| 15 | 40,000 | 2,000 | 2.5% | 1,063 | 0.988 |
| 15 | 40,000 | 2,000 | 5% | 2,002 | 0.996 |
| 15 | 40,000 | 2,000 | 10% | 4,267 | 0.998 |
| 15 | 40,000 | 4,000 | 1% | 456 | 0.951 |
| 15 | 40,000 | 4,000 | 2.5% | 1,054 | 0.983 |
| 15 | 40,000 | 4,000 | 5% | 2,041 | 0.997 |
| 15 | 40,000 | 4,000 | 10% | 4,055 | 0.999 |
| 15 | 80,000 | 500 | 1% | 805 | 0.963 |
| 15 | 80,000 | 500 | 2.5% | 2,011 | 0.964 |
| 15 | 80,000 | 500 | 5% | 4,221 | 0.965 |
| 15 | 80,000 | 500 | 10% | 8,506 | 0.965 |
| 15 | 80,000 | 1,000 | 1% | 812 | 0.978 |
| 15 | 80,000 | 1,000 | 2.5% | 2,067 | 0.990 |
| 15 | 80,000 | 1,000 | 5% | 4,010 | 0.990 |
| 15 | 80,000 | 1,000 | 10% | 8,701 | 0.991 |
| 15 | 80,000 | 2,000 | 1% | 824 | 0.973 |
| 15 | 80,000 | 2,000 | 2.5% | 2,108 | 0.995 |
| 15 | 80,000 | 2,000 | 5% | 4,213 | 0.996 |
| 15 | 80,000 | 2,000 | 10% | 8,279 | 0.997 |
| 15 | 80,000 | 4,000 | 1% | 865 | 0.974 |
| 15 | 80,000 | 4,000 | 2.5% | 2,084 | 0.995 |
| 15 | 80,000 | 4,000 | 5% | 4,032 | 0.998 |
| 15 | 80,000 | 4,000 | 10% | 8,099 | 0.999 |
| 30 | 20,000 | 500 | 1% | 209 | 0.942 |
| 30 | 20,000 | 500 | 2.5% | 502 | 0.973 |
| 30 | 20,000 | 500 | 5% | 1,006 | 0.976 |
| 30 | 20,000 | 500 | 10% | 2,060 | 0.976 |
| 30 | 20,000 | 1,000 | 1% | 216 | 0.934 |
| 30 | 20,000 | 1,000 | 2.5% | 508 | 0.970 |
| 30 | 20,000 | 1,000 | 5% | 1,029 | 0.989 |
| 30 | 20,000 | 1,000 | 10% | 2,072 | 0.991 |
| 30 | 20,000 | 2,000 | 1% | 225 | 0.911 |
| 30 | 20,000 | 2,000 | 2.5% | 527 | 0.965 |
| 30 | 20,000 | 2,000 | 5% | 1,003 | 0.989 |
| 30 | 20,000 | 2,000 | 10% | 2,027 | 0.997 |
| 30 | 20,000 | 4,000 | 1% | 204 | 0.913 |
| 30 | 20,000 | 4,000 | 2.5% | 523 | 0.966 |
| 30 | 20,000 | 4,000 | 5% | 1,000 | 0.987 |
| 30 | 20,000 | 4,000 | 10% | 2,105 | 0.997 |
| 30 | 40,000 | 500 | 1% | 422 | 0.955 |
| 30 | 40,000 | 500 | 2.5% | 1,014 | 0.968 |
| 30 | 40,000 | 500 | 5% | 2,008 | 0.968 |
| 30 | 40,000 | 500 | 10% | 4,077 | 0.968 |
| 30 | 40,000 | 1,000 | 1% | 408 | 0.950 |
| 30 | 40,000 | 1,000 | 2.5% | 1,007 | 0.986 |
| 30 | 40,000 | 1,000 | 5% | 2,118 | 0.988 |
| 30 | 40,000 | 1,000 | 10% | 4,043 | 0.988 |
| 30 | 40,000 | 2,000 | 1% | 424 | 0.953 |
| 30 | 40,000 | 2,000 | 2.5% | 1,020 | 0.986 |
| 30 | 40,000 | 2,000 | 5% | 2,010 | 0.996 |
| 30 | 40,000 | 2,000 | 10% | 4,068 | 0.997 |
| 30 | 40,000 | 4,000 | 1% | 436 | 0.953 |
| 30 | 40,000 | 4,000 | 2.5% | 1,065 | 0.986 |
| 30 | 40,000 | 4,000 | 5% | 2,109 | 0.996 |
| 30 | 40,000 | 4,000 | 10% | 4,078 | 0.999 |
| 30 | 80,000 | 500 | 1% | 820 | 0.974 |
| 30 | 80,000 | 500 | 2.5% | 2,129 | 0.974 |
| 30 | 80,000 | 500 | 5% | 4,058 | 0.974 |
| 30 | 80,000 | 1,000 | 1% | 817 | 0.985 |
| 30 | 80,000 | 1,000 | 2.5% | 2,110 | 0.990 |
| 30 | 80,000 | 1,000 | 5% | 4,246 | 0.990 |
| 30 | 80,000 | 1,000 | 10% | 8,028 | 0.990 |
| 30 | 80,000 | 2,000 | 1% | 818 | 0.983 |
| 30 | 80,000 | 2,000 | 2.5% | 2,042 | 0.996 |
| 30 | 80,000 | 2,000 | 5% | 4,169 | 0.997 |
| 30 | 80,000 | 2,000 | 10% | 8,208 | 0.997 |
| 30 | 80,000 | 4,000 | 1% | 866 | 0.981 |
| 30 | 80,000 | 4,000 | 2.5% | 2,008 | 0.995 |
| 30 | 80,000 | 4,000 | 5% | 4,046 | 0.998 |
| 30 | 80,000 | 4,000 | 10% | 8,157 | 0.999 |
| 60 | 20,000 | 500 | 1% | 216 | 0.858 |
| 60 | 20,000 | 500 | 2.5% | 503 | 0.950 |
| 60 | 20,000 | 500 | 5% | 1,015 | 0.954 |
| 60 | 20,000 | 500 | 10% | 2,021 | 0.954 |
| 60 | 20,000 | 1,000 | 1% | 231 | 0.875 |
| 60 | 20,000 | 1,000 | 2.5% | 510 | 0.953 |
| 60 | 20,000 | 1,000 | 5% | 1,001 | 0.978 |
| 60 | 20,000 | 1,000 | 10% | 2,042 | 0.980 |
| 60 | 20,000 | 2,000 | 1% | 222 | 0.890 |
| 60 | 20,000 | 2,000 | 2.5% | 503 | 0.941 |
| 60 | 20,000 | 2,000 | 5% | 1,054 | 0.976 |
| 60 | 20,000 | 2,000 | 10% | 2,000 | 0.993 |
| 60 | 20,000 | 4,000 | 1% | 212 | 0.857 |
| 60 | 20,000 | 4,000 | 2.5% | 518 | 0.939 |
| 60 | 20,000 | 4,000 | 5% | 1,008 | 0.971 |
| 60 | 20,000 | 4,000 | 10% | 2,006 | 0.992 |
| 60 | 40,000 | 500 | 1% | 411 | 0.936 |
| 60 | 40,000 | 500 | 2.5% | 1,016 | 0.942 |
| 60 | 40,000 | 500 | 5% | 2,033 | 0.942 |
| 60 | 40,000 | 1,000 | 1% | 401 | 0.928 |
| 60 | 40,000 | 1,000 | 2.5% | 1,011 | 0.973 |
| 60 | 40,000 | 1,000 | 5% | 2,122 | 0.975 |
| 60 | 40,000 | 1,000 | 10% | 4,027 | 0.975 |
| 60 | 40,000 | 2,000 | 1% | 420 | 0.942 |
| 60 | 40,000 | 2,000 | 2.5% | 1,033 | 0.967 |
| 60 | 40,000 | 2,000 | 5% | 2,036 | 0.988 |
| 60 | 40,000 | 2,000 | 10% | 4,007 | 0.990 |
| 60 | 40,000 | 4,000 | 1% | 434 | 0.939 |
| 60 | 40,000 | 4,000 | 2.5% | 1,057 | 0.966 |
| 60 | 40,000 | 4,000 | 5% | 2,030 | 0.986 |
| 60 | 40,000 | 4,000 | 10% | 4,097 | 0.998 |
| 60 | 80,000 | 500 | 1% | 804 | 0.945 |
| 60 | 80,000 | 500 | 2.5% | 2,021 | 0.945 |
| 60 | 80,000 | 1,000 | 1% | 810 | 0.956 |
| 60 | 80,000 | 1,000 | 2.5% | 2,043 | 0.968 |
| 60 | 80,000 | 1,000 | 5% | 4,095 | 0.968 |
| 60 | 80,000 | 2,000 | 1% | 853 | 0.936 |
| 60 | 80,000 | 2,000 | 2.5% | 2,013 | 0.984 |
| 60 | 80,000 | 2,000 | 5% | 4,028 | 0.986 |
| 60 | 80,000 | 2,000 | 10% | 8,317 | 0.986 |
| 60 | 80,000 | 4,000 | 1% | 878 | 0.935 |
| 60 | 80,000 | 4,000 | 2.5% | 2,025 | 0.982 |
| 60 | 80,000 | 4,000 | 5% | 4,036 | 0.995 |
| 60 | 80,000 | 4,000 | 10% | 8,116 | 0.996 |

# **Table S3.**

Output of the linear mixed effect analysis model, which aims to estimate the relationship between the error of the Lasso approximation and the number of positions, number of sequences, sequence divergence, percentage of constant sites and the alpha parameter. Analysis was performed using training size ($\eta$ = 4,000) and ζ = 5% of the positions.

| **Fixed effect** | **Coefficient** | **P-value** |
| --- | --- | --- |
| (Intercept) | -3.68 | 0.00 |
| Number of positions | 0.00 | 0.00 |
| Number of sequences | 0.03 | 0.0001 |
| Divergence | -0.02 | 0.60 |
| Percentage of constant sites | -1.16 | 0.14 |
| Alpha | -1.23 | 0.035 |

# **Table S4.**

Performance of the Lasso methodology on six empirical amino-acid MSAs trimmed to 20,000 positions and 30 taxa under WAG+G, JTT+G and LG +G models.

| **Dataset name** | **Sample fraction** | **WAG test** $\boldsymbol{r}^{\mathbf{2}}$ | **JTT test** $\boldsymbol{r}^{\mathbf{2}}$ | **LG test** $\boldsymbol{r}^{\mathbf{2}}$ |
| --- | --- | --- | --- | --- |
| MisoA2 | 0.01 | 0.927 | 0.910 | 0.910 |
| MisoA2 | 0.025 | 0.942 | 0.944 | 0.944 |
| MisoA2 | 0.05 | 0.971 | 0.976 | 0.976 |
| MisoA2 | 0.1 | 0.992 | 0.994 | 0.994 |
| NagyA1 | 0.01 | 0.978 | 0.983 | 0.983 |
| NagyA1 | 0.025 | 0.983 | 0.983 | 0.983 |
| NagyA1 | 0.05 | 0.995 | 0.996 | 0.996 |
| NagyA1 | 0.1 | 0.999 | 0.999 | 0.999 |
| ShenA9 | 0.01 | 0.965 | 0.965 | 0.965 |
| ShenA9 | 0.025 | 0.967 | 0.971 | 0.971 |
| ShenA9 | 0.05 | 0.985 | 0.983 | 0.983 |
| ShenA9 | 0.1 | 0.997 | 0.997 | 0.997 |
| StruA5 | 0.01 | 0.834 | 0.837 | 0.837 |
| StruA5 | 0.025 | 0.916 | 0.900 | 0.900 |
| StruA5 | 0.05 | 0.968 | 0.966 | 0.966 |
| StruA5 | 0.1 | 0.991 | 0.990 | 0.990 |
| WickA3 | 0.01 | 0.924 | 0.940 | 0.940 |
| WickA3 | 0.025 | 0.953 | 0.965 | 0.965 |
| WickA3 | 0.05 | 0.986 | 0.988 | 0.988 |
| WickA3 | 0.1 | 0.997 | 0.997 | 0.997 |
| YangA8 | 0.01 | 0.913 | 0.915 | 0.915 |
| YangA8 | 0.025 | 0.966 | 0.971 | 0.971 |
| YangA8 | 0.05 | 0.987 | 0.990 | 0.990 |
| YangA8 | 0.1 | 0.997 | 0.997 | 0.997 |

Performance of the Lasso methodology under the GTR+G model on four DNA MSAs.

| **Dataset name** | **Sample fraction** | **WAG test** $\boldsymbol{r}^{\mathbf{2}}$ |
| --- | --- | --- |
| MisoD2a | 0.01 | 0.482 |
| MisoD2a | 0.025 | 0.915 |
| MisoD2a | 0.05 | 0.956 |
| MisoD2a | 0.1 | 0.973 |
| PrumD6 | 0.01 | 0.963 |
| PrumD6 | 0.025 | 0.990 |
| PrumD6 | 0.05 | 0.993 |
| PrumD6 | 0.1 | 0.995 |
| WickD3a | 0.01 | 0.798 |
| WickD3a | 0.025 | 0.925 |
| WickD3a | 0.05 | 0.946 |
| WickD3a | 0.1 | 0.984 |
| WickD3b | 0.01 | 0.937 |
| WickD3b | 0.025 | 0.967 |
| WickD3b | 0.05 | 0.984 |
| WickD3b | 0.1 | 0.996 |

# **Table S5.**

Comparison of the Lasso methodology to random sampling and selection of fastest evolving positions on six empirical amino-acid MSAs trimmed to 20,000 positions and 30. Shown is the square Pearson correlation coefficient ($r^{2}$) obtained on a test set of 100 random trees

| **Dataset name** | **Sample fraction** | **Lasso- test** $\boldsymbol{r}^{\mathbf{2}}$ | **Random- test** $\boldsymbol{r}^{\mathbf{2}}$ | **Fast-evolving sites - test** $\boldsymbol{r}^{\mathbf{2}}$ |
| --- | --- | --- | --- | --- |
| YangA8 | 0.01 | 0.913 | 0.831 | 0.471 |
| YangA8 | 0.025 | 0.966 | 0.920 | 0.595 |
| YangA8 | 0.05 | 0.987 | 0.966 | 0.708 |
| YangA8 | 0.1 | 0.997 | 0.983 | 0.862 |
| MisoA2 | 0.01 | 0.927 | 0.774 | 0.491 |
| MisoA2 | 0.025 | 0.942 | 0.915 | 0.397 |
| MisoA2 | 0.05 | 0.971 | 0.947 | 0.517 |
| MisoA2 | 0.1 | 0.992 | 0.980 | 0.670 |
| NagyA1 | 0.01 | 0.978 | 0.921 | 0.815 |
| NagyA1 | 0.025 | 0.983 | 0.966 | 0.886 |
| NagyA1 | 0.05 | 0.995 | 0.984 | 0.910 |
| NagyA1 | 0.1 | 0.999 | 0.992 | 0.934 |
| ShenA9 | 0.01 | 0.965 | 0.936 | 0.524 |
| ShenA9 | 0.025 | 0.967 | 0.973 | 0.600 |
| ShenA9 | 0.05 | 0.985 | 0.986 | 0.636 |
| ShenA9 | 0.1 | 0.997 | 0.994 | 0.709 |
| StruA5 | 0.01 | 0.834 | 0.425 | 0.638 |
| StruA5 | 0.025 | 0.916 | 0.633 | 0.807 |
| StruA5 | 0.05 | 0.968 | 0.822 | 0.865 |
| StruA5 | 0.1 | 0.991 | 0.905 | 0.936 |
| WickA3 | 0.01 | 0.924 | 0.885 | 0.473 |
| WickA3 | 0.025 | 0.953 | 0.954 | 0.531 |
| WickA3 | 0.05 | 0.986 | 0.978 | 0.681 |
| WickA3 | 0.1 | 0.997 | 0.989 | 0.808 |

# **Table S6.**

Each of the six panels corresponds to a different empirical MSA. Shown is the performance of the Standard search, Lasso-only search and Two-phase search on 18 amino-acid empirical MSAs, each with a total of 80,000 positions (six datasets, trimmed to include either 15, 30, or 60 sequences), with searches starting from three random points.

| a | Number of sequences |
| --- | --- |
| b | Starting tree index |
| c | Standard search final LL |
| d | Standard search SPR moves |
| e | Standard search CPU time |
| f | Only-Lasso search final LL |
| g | Only-Lasso search SPR moves |
| h | Only-Lasso search CPU time |
| i | Two-phase search final LL |
| j | Two-phase search SPR moves |
| k | Two-phase search CPU time |
| l | Training-phase CPU time |

## **MisoA2 dataset**

| **a** | **b** | **c** | **d** | **e** | **f** | **g** | **h** | **i** | **j** | **k** | **l** |
| --- | --- | --- | --- | --- | --- | --- | --- | --- | --- | --- | --- |
| 15 | 1 | -866,452.84 | 55 | 21,038.99 | -866,207.64 | 71 | 922.35 | -866,207.64 | 72 | 3,783.72 | 6,763.32 |
| 15 | 2 | -866,452.84 | 58 | 30,985.71 | -866,474.80 | 66 | 749.21 | -866,474.80 | 67 | 3,627.70 | 6,763.32 |
| 15 | 3 | -866,452.84 | 44 | 23,890.14 | -866,207.64 | 49 | 1,062.40 | -866,207.64 | 50 | 3,972.61 | 6,763.32 |
| 30 | 1 | -1,471,493.28 | 124 | 95,620.97 | -1,471,512.68 | 141 | 2,707.63 | -1,471,512.68 | 142 | 5,553.01 | 12,115.71 |
| 30 | 2 | -1,471,448.45 | 168 | 151,626.30 | -1,472,232.54 | 146 | 3,892.55 | -1,471,448.45 | 150 | 7,897.63 | 12,115.71 |
| 30 | 3 | -1,471,448.45 | 107 | 83,902.88 | -1,471,448.45 | 143 | 3,994.22 | -1,471,448.45 | 144 | 6,632.86 | 12,115.71 |
| 60 | 1 | -2,474,330.14 | 397 | 688,372.74 | -2,474,114.62 | 452 | 14,636.19 | -2,474,104.88 | 454 | 20,464.12 | 21,335.12 |
| 60 | 2 | -2,474,147.71 | 422 | 544,313.93 | -2,474,538.48 | 476 | 12,677.97 | -2,474,421.98 | 479 | 18,711.13 | 21,335.12 |
| 60 | 3 | -2,474,113.73 | 452 | 889,696.75 | -2,474,758.31 | 409 | 14,031.87 | -2,474,019.23 | 418 | 28,821.66 | 21,335.12 |

## **NagyA1 dataset**

| **a** | **b** | **c** | **d** | **e** | **f** | **g** | **h** | **i** | **j** | **k** | **l** |
| --- | --- | --- | --- | --- | --- | --- | --- | --- | --- | --- | --- |
| 15 | 1 | -1,167,998.20 | 42 | 23,966.49 | -1,167,950.93 | 37 | 933.62 | -1,167,858.52 | 39 | 5,071.33 | 8,376.71 |
| 15 | 2 | -1,167,858.52 | 49 | 24,101.52 | -1,167,947.87 | 49 | 1,083.84 | -1,167,947.87 | 50 | 5,052.45 | 8,376.71 |
| 15 | 3 | -1,167,858.52 | 37 | 38,083.40 | -1,167,947.87 | 28 | 1,019.31 | -1,167,858.52 | 30 | 8,853.65 | 8,376.71 |
| 30 | 1 | -1,926,473.86 | 165 | 85,794.84 | -1,926,871.78 | 169 | 1,823.36 | -1,926,138.75 | 177 | 11,971.10 | 12,210.65 |
| 30 | 2 | -1,925,986.06 | 167 | 133,817.96 | -1,926,169.64 | 153 | 2,847.76 | -1,925,950.83 | 157 | 6,208.53 | 12,210.65 |
| 30 | 3 | -1,925,986.06 | 154 | 102,948.01 | -1,926,868.15 | 164 | 1,540.29 | -1,926,660.28 | 167 | 6,637.61 | 12,210.65 |
| 60 | 1 | -3,037,001.31 | 424 | 537,257.57 | -3,037,050.47 | 433 | 14,490.01 | -3,036,893.99 | 437 | 20,804.23 | 22,134.90 |
| 60 | 2 | -3,036,657.44 | 418 | 731,120.60 | -3,037,247.18 | 434 | 13,101.24 | -3,037,159.65 | 436 | 17,765.95 | 22,134.90 |
| 60 | 3 | -3,036,310.96 | 404 | 273,143.30 | -3,036,720.90 | 489 | 12,042.05 | -3,036,633.03 | 491 | 16,646.18 | 22,134.90 |

## **ShenA9 dataset**

| **a** | **b** | **c** | **d** | **e** | **f** | **g** | **h** | **i** | **j** | **k** | **l** |
| --- | --- | --- | --- | --- | --- | --- | --- | --- | --- | --- | --- |
| 15 | 1 | -1,718,303.66 | 36 | 21,412.26 | -1,718,303.67 | 58 | 956.45 | -1,718,303.67 | 59 | 5,995.22 | 9,796.76 |
| 15 | 2 | -1,718,303.66 | 33 | 25,830.26 | -1,718,303.67 | 68 | 722.62 | -1,718,303.67 | 69 | 5,533.52 | 9,796.76 |
| 15 | 3 | -1,718,303.66 | 40 | 27,843.08 | -1,718,303.67 | 51 | 1,084.72 | -1,718,303.67 | 52 | 6,247.26 | 9,796.76 |
| 30 | 1 | -2,935,390.39 | 151 | 91,707.24 | -2,934,598.97 | 141 | 1,145.80 | -2,934,598.97 | 142 | 3,664.62 | 15,688.80 |
| 30 | 2 | -2,934,462.49 | 131 | 64,344.03 | -2,934,701.11 | 152 | 1,198.02 | -2,934,462.49 | 156 | 4,560.01 | 15,688.80 |
| 30 | 3 | -2,934,462.49 | 148 | 111,962.16 | -2,934,564.77 | 160 | 1,955.83 | -2,934,462.49 | 163 | 5,039.60 | 15,688.80 |
| 60 | 1 | -4,854,781.65 | 486 | 593,643.62 | -4,854,993.41 | 418 | 10,790.42 | -4,854,698.91 | 421 | 16,212.02 | 23,559.21 |
| 60 | 2 | -4,854,563.13 | 393 | 414,946.58 | -4,856,190.24 | 443 | 10,463.10 | -4,855,634.33 | 448 | 17,025.79 | 23,559.21 |
| 60 | 3 | -4,854,478.73 | 407 | 452,366.83 | -4,855,862.25 | 429 | 8,308.06 | -4,855,862.25 | 430 | 13,006.19 | 23,559.21 |

## **StruA5 dataset**

| **a** | **b** | **c** | **d** | **e** | **f** | **g** | **h** | **i** | **j** | **k** | **l** |
| --- | --- | --- | --- | --- | --- | --- | --- | --- | --- | --- | --- |
| 15 | 1 | -549,467.35 | 73 | 63,272.97 | -549,475.61 | 50 | 1,939.89 | -549,467.35 | 53 | 6,236.98 | 6,220.80 |
| 15 | 2 | -549,469.74 | 47 | 52,626.82 | -549,477.80 | 41 | 1,388.07 | -549,467.35 | 44 | 5,648.62 | 6,220.80 |
| 15 | 3 | -549,469.74 | 51 | 65,621.73 | -549,475.61 | 51 | 1,479.09 | -549,469.74 | 53 | 4,646.69 | 6,220.80 |
| 30 | 1 | -796,491.26 | 190 | 496,006.21 | -796,676.35 | 213 | 13,936.53 | -796,636.67 | 215 | 22,585.37 | 24,405.06 |
| 30 | 2 | -796,612.64 | 153 | 481,385.55 | -796,789.80 | 160 | 10,438.70 | -796,617.38 | 170 | 42,661.29 | 24,405.06 |
| 30 | 3 | -796,390.06 | 147 | 644,596.92 | -796,753.10 | 206 | 14,833.57 | -796,589.42 | 215 | 38,461.57 | 24,405.06 |
| 60 | 1 | -1,359,325.01 | 437 | 3,250,373.08 | -1,359,465.78 | 421 | 48,474.89 | -1,359,382.95 | 429 | 106,586.56 | 50,898.09 |
| 60 | 2 | -1,359,157.83 | 431 | 2,706,634.06 | -1,359,433.14 | 443 | 50,529.82 | -1,359,325.76 | 452 | 107,490.84 | 50,898.09 |
| 60 | 3 | -1,359,239.91 | 430 | 1,978,530.54 | -1,359,538.49 | 461 | 46,082.50 | -1,359,447.85 | 466 | 103,406.53 | 50,898.09 |

## **WickA3 dataset**

| **a** | **b** | **c** | **d** | **e** | **f** | **g** | **h** | **i** | **j** | **k** | **l** |
| --- | --- | --- | --- | --- | --- | --- | --- | --- | --- | --- | --- |
| 15 | 1 | -1,072,297.74 | 51 | 64,594.74 | -1,072,327.81 | 47 | 1,571.83 | -1,072,297.74 | 50 | 15,148.68 | 8,912.40 |
| 15 | 2 | -1,072,297.63 | 53 | 38,931.90 | -1,072,297.66 | 46 | 1,341.61 | -1,072,297.66 | 47 | 8,069.30 | 8,912.40 |
| 15 | 3 | -1,072,297.74 | 72 | 61,216.48 | -1,072,327.77 | 53 | 2,346.10 | -1,072,297.64 | 55 | 15,297.91 | 8,912.40 |
| 30 | 1 | -1,731,365.83 | 155 | 419,442.32 | -1,731,261.43 | 221 | 21,499.60 | -1,730,941.88 | 225 | 42,293.34 | 36,041.00 |
| 30 | 2 | -1,731,142.49 | 122 | 266,283.36 | -1,731,116.00 | 167 | 11,901.57 | -1,731,113.75 | 169 | 26,326.68 | 36,041.00 |
| 30 | 3 | -1,730,991.08 | 233 | 309,846.36 | -1,731,261.43 | 230 | 9,980.99 | -1,730,941.88 | 234 | 27,722.05 | 36,041.00 |
| 60 | 1 | -3,071,438.92 | 560 | 2,497,772.57 | -3,072,039.28 | 449 | 37,427.47 | -3,070,880.28 | 459 | 140,737.36 | 76,369.67 |
| 60 | 2 | -3,071,860.59 | 444 | 1,788,004.60 | -3,072,096.26 | 440 | 40,536.02 | -3,072,031.72 | 442 | 81,296.84 | 76,369.67 |
| 60 | 3 | -3,071,476.96 | 487 | 1,580,629.77 | -3,071,270.27 | 489 | 30,038.48 | -3,071,180.40 | 491 | 67,772.19 | 76,369.67 |

## **YangA8 dataset**

| **a** | **b** | **c** | **d** | **e** | **f** | **g** | **h** | **i** | **j** | **k** | **l** |
| --- | --- | --- | --- | --- | --- | --- | --- | --- | --- | --- | --- |
| 15 | 1 | -750,070.91 | 42 | 22,720.79 | -750,070.91 | 59 | 693.08 | -750,070.91 | 60 | 3,647.27 | 6,910.45 |
| 15 | 2 | -750,070.91 | 45 | 23,291.82 | -750,070.91 | 58 | 578.11 | -750,070.91 | 59 | 3,853.27 | 6,910.45 |
| 15 | 3 | -750,070.91 | 46 | 32,279.60 | -750,070.91 | 50 | 818.92 | -750,070.91 | 51 | 3,846.87 | 6,910.45 |
| 30 | 1 | -1,173,509.25 | 120 | 52,410.63 | -1,173,521.28 | 114 | 1,596.49 | -1,173,395.46 | 118 | 5,453.23 | 11,832.95 |
| 30 | 2 | -1,173,387.11 | 158 | 86,173.05 | -1,173,521.28 | 168 | 1,496.51 | -1,173,387.11 | 173 | 6,551.30 | 11,832.95 |
| 30 | 3 | -1,173,722.74 | 126 | 53,938.57 | -1,173,521.28 | 134 | 2,240.76 | -1,173,387.11 | 139 | 6,625.73 | 11,832.95 |
| 60 | 1 | -2,087,519.13 | 415 | 442,651.03 | -2,087,368.87 | 419 | 8,183.85 | -2,087,361.24 | 421 | 13,734.55 | 23,733.75 |
| 60 | 2 | -2,087,135.48 | 399 | 482,261.79 | -2,087,154.54 | 419 | 9,867.43 | -2,087,015.21 | 424 | 16,794.82 | 23,733.75 |
| 60 | 3 | -2,087,193.87 | 332 | 470,969.74 | -2,087,064.76 | 398 | 10,710.77 | -2,087,015.21 | 400 | 15,666.27 | 23,733.75 |

**Alignment-size = 80,0000**

**
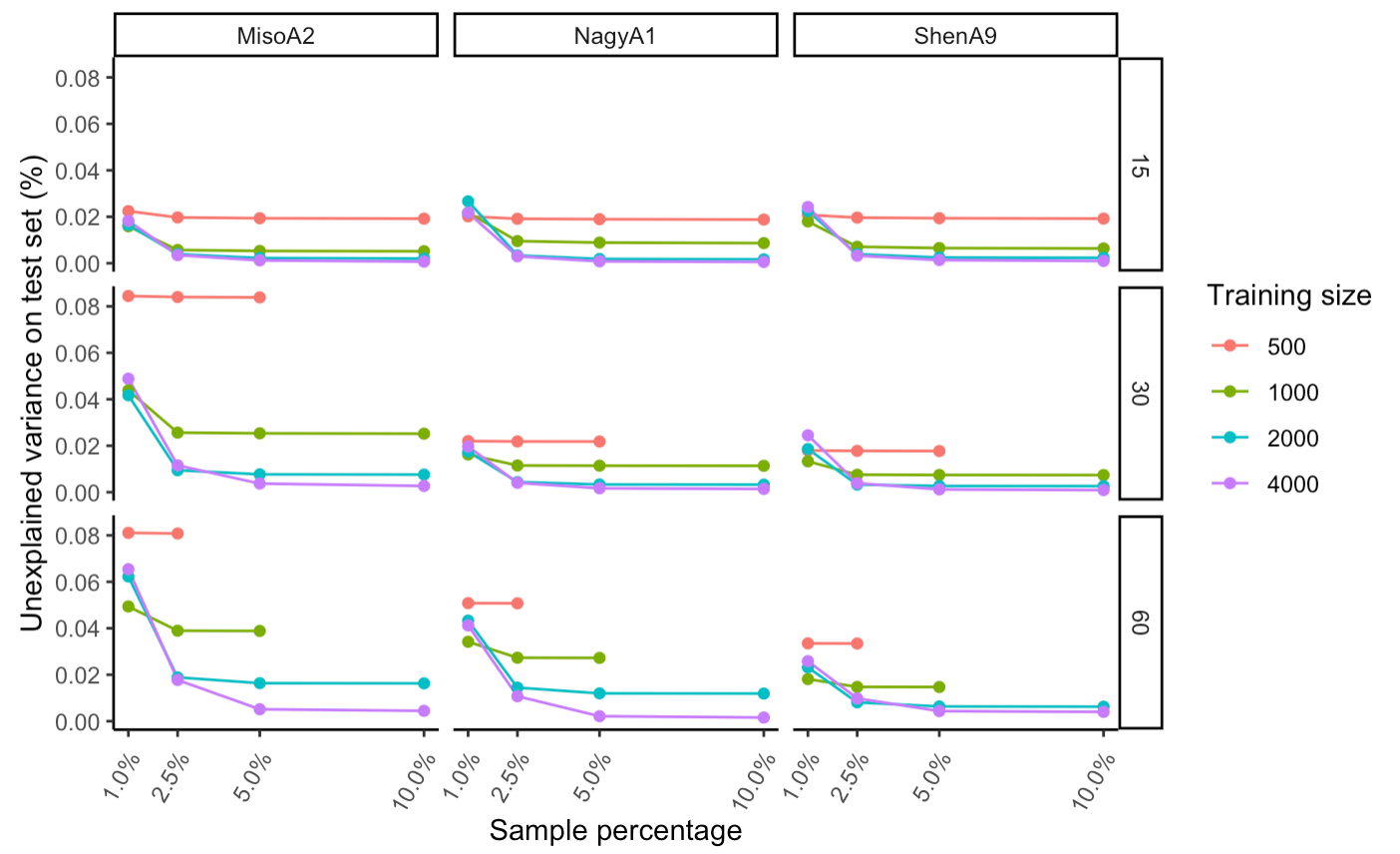
**

**Alignment-size = 40,0000**

**
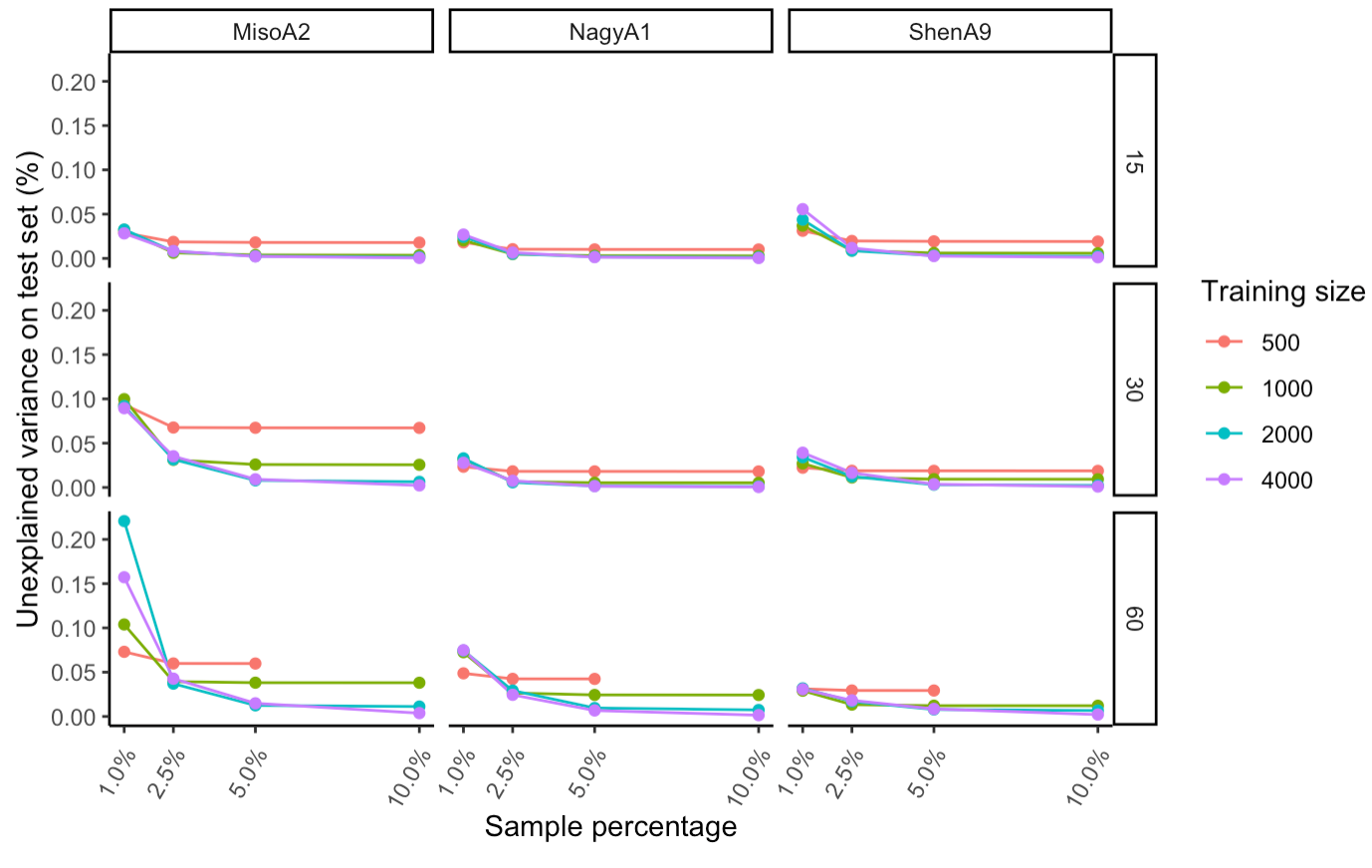
**

**Alignment-size = 20,0000**

**
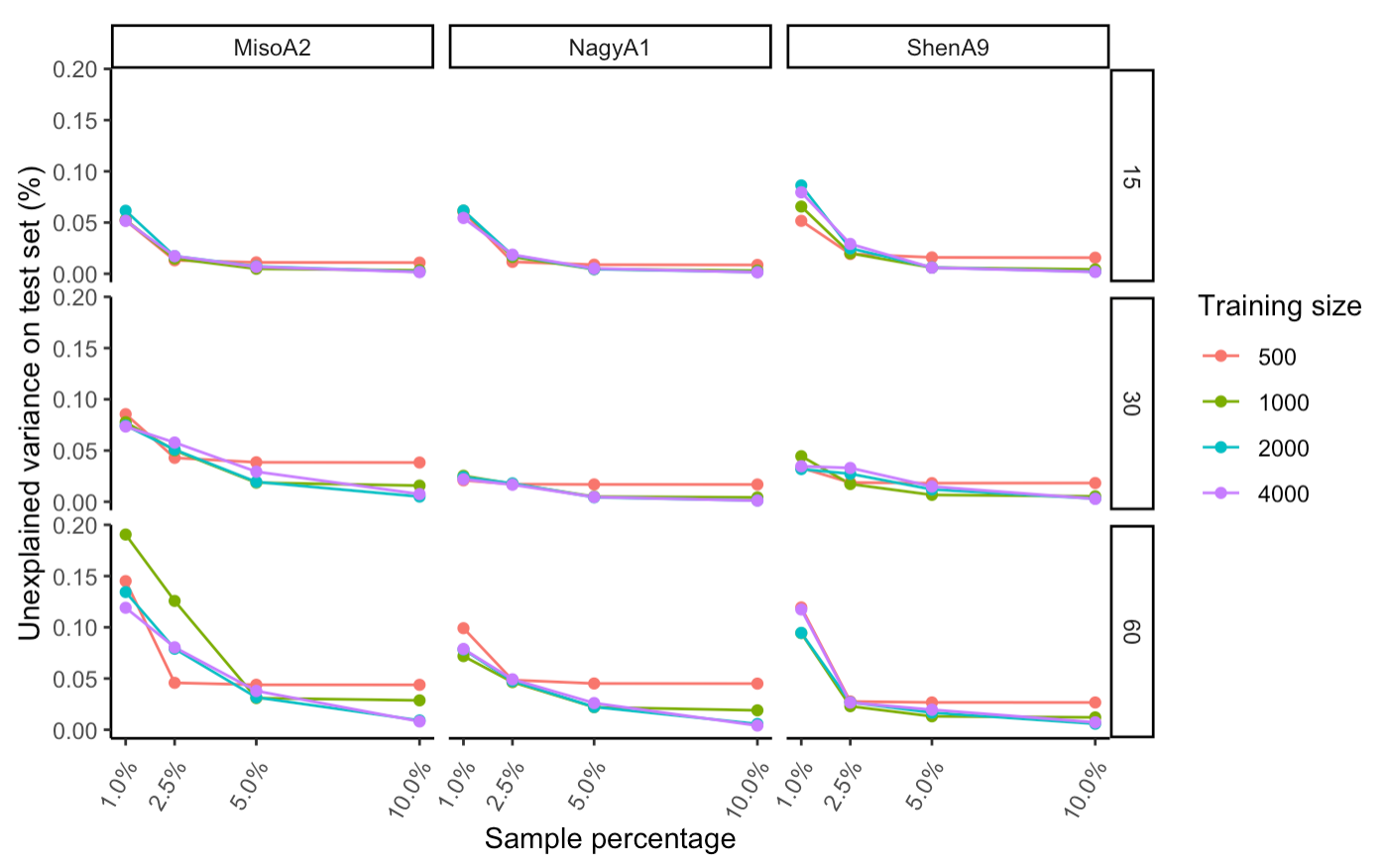
**

**Alignment-size = 80,0000**

**
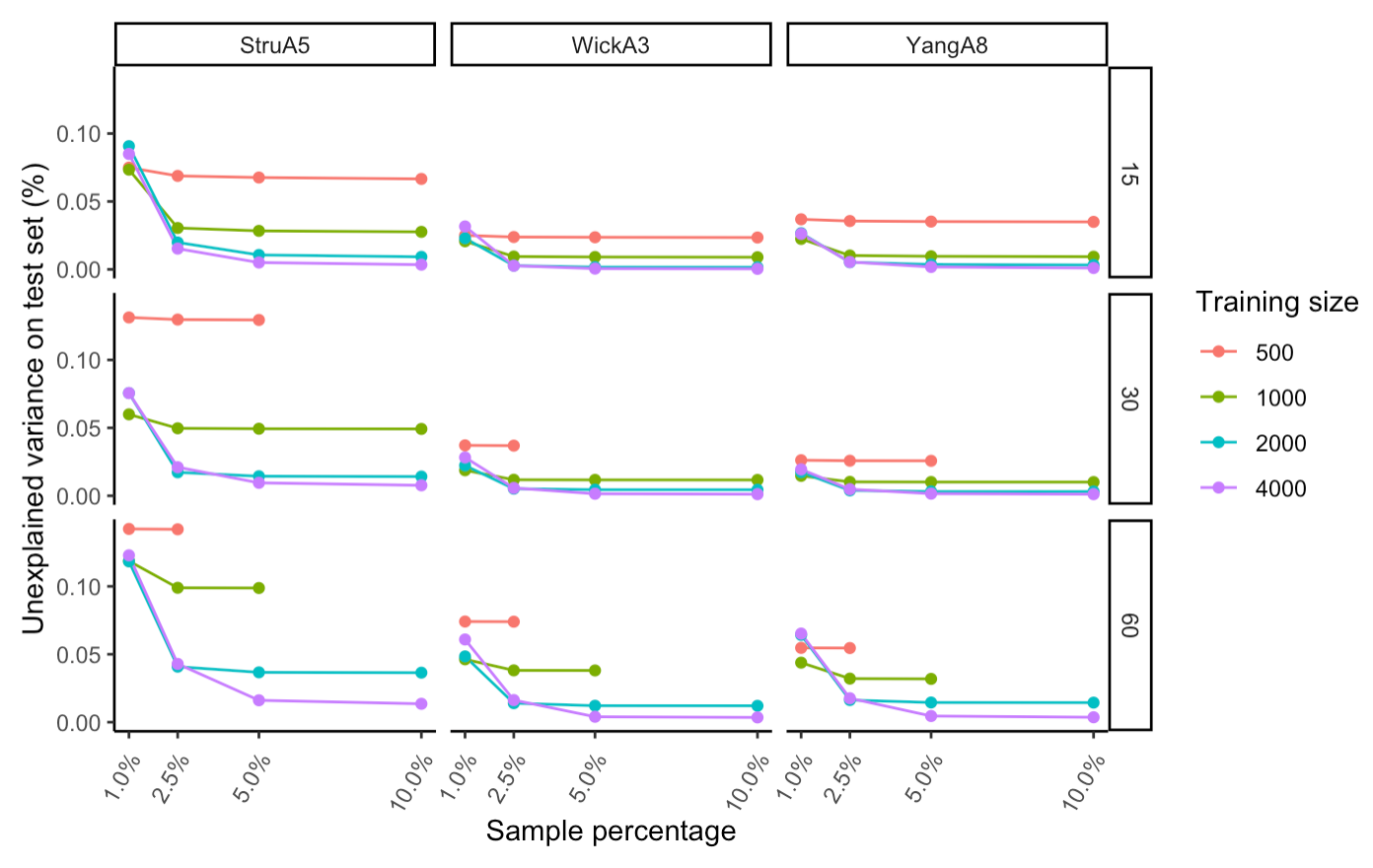
**

**Alignment-size = 40,0000**

**
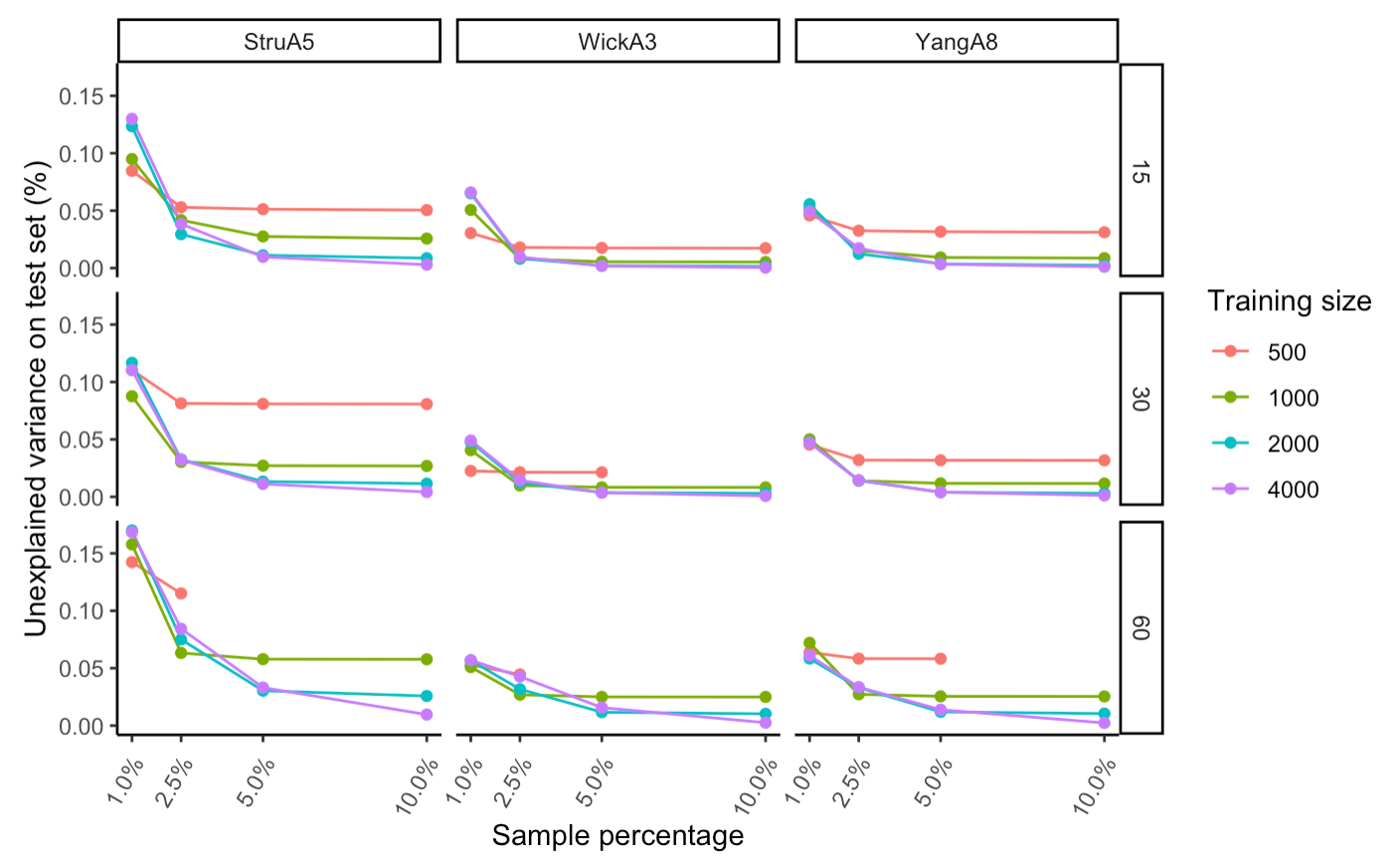
**

**Alignment-size = 20,0000**

**
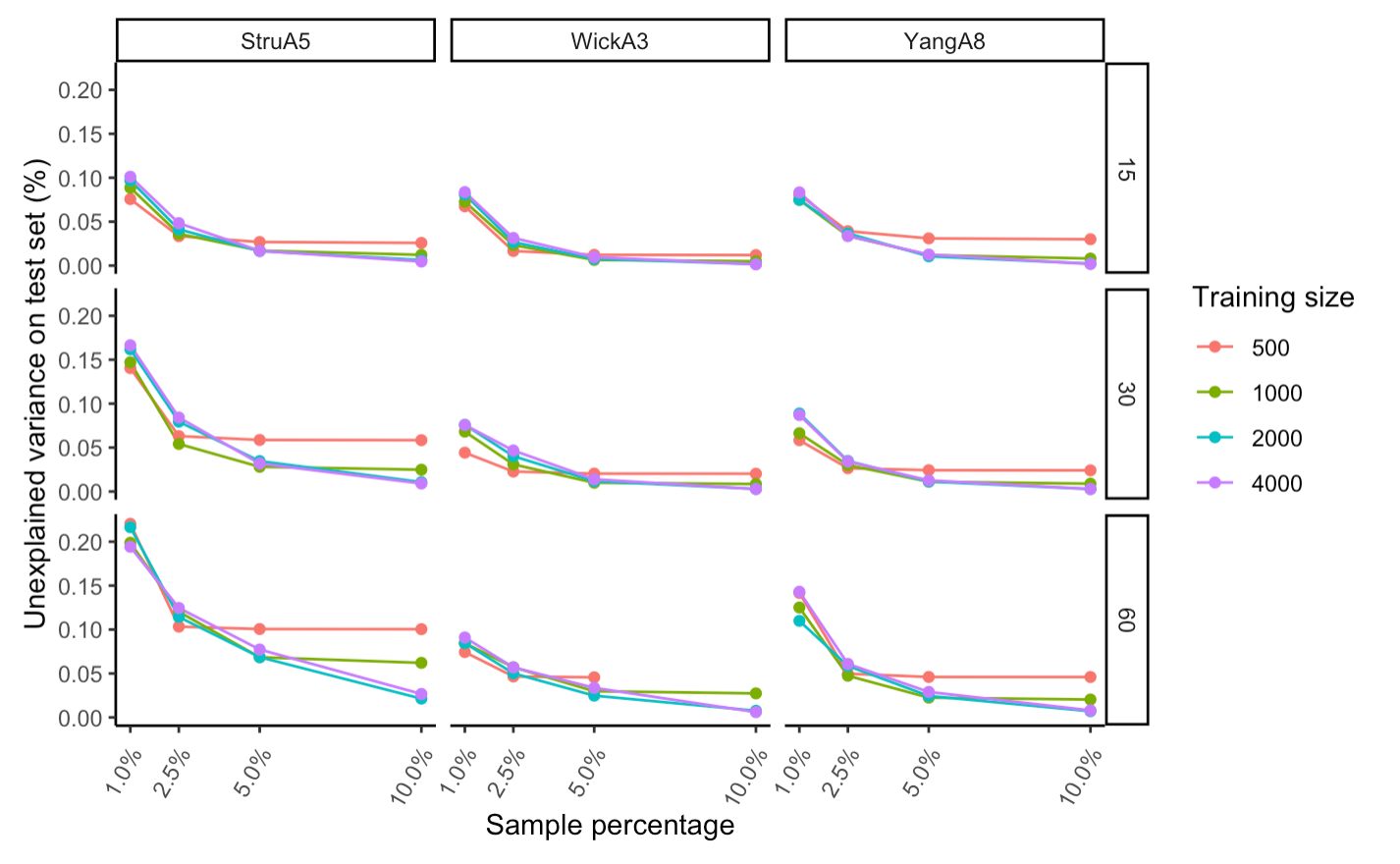
**

# **Figure S1.**

Each of the six panels corresponds to three empirical datasets trimmed to a specific alignment length (either 20,000, 40,000, or 80,000 residues). Shown are line plot of the percentage of unexplained variance ($1-r^{2}$) for four values of training size, four values of sample percentage and four values of number of sequences. The performance was evaluated on 100 test trees, i.e., trees that were not used for training.

**ShenA9**

**
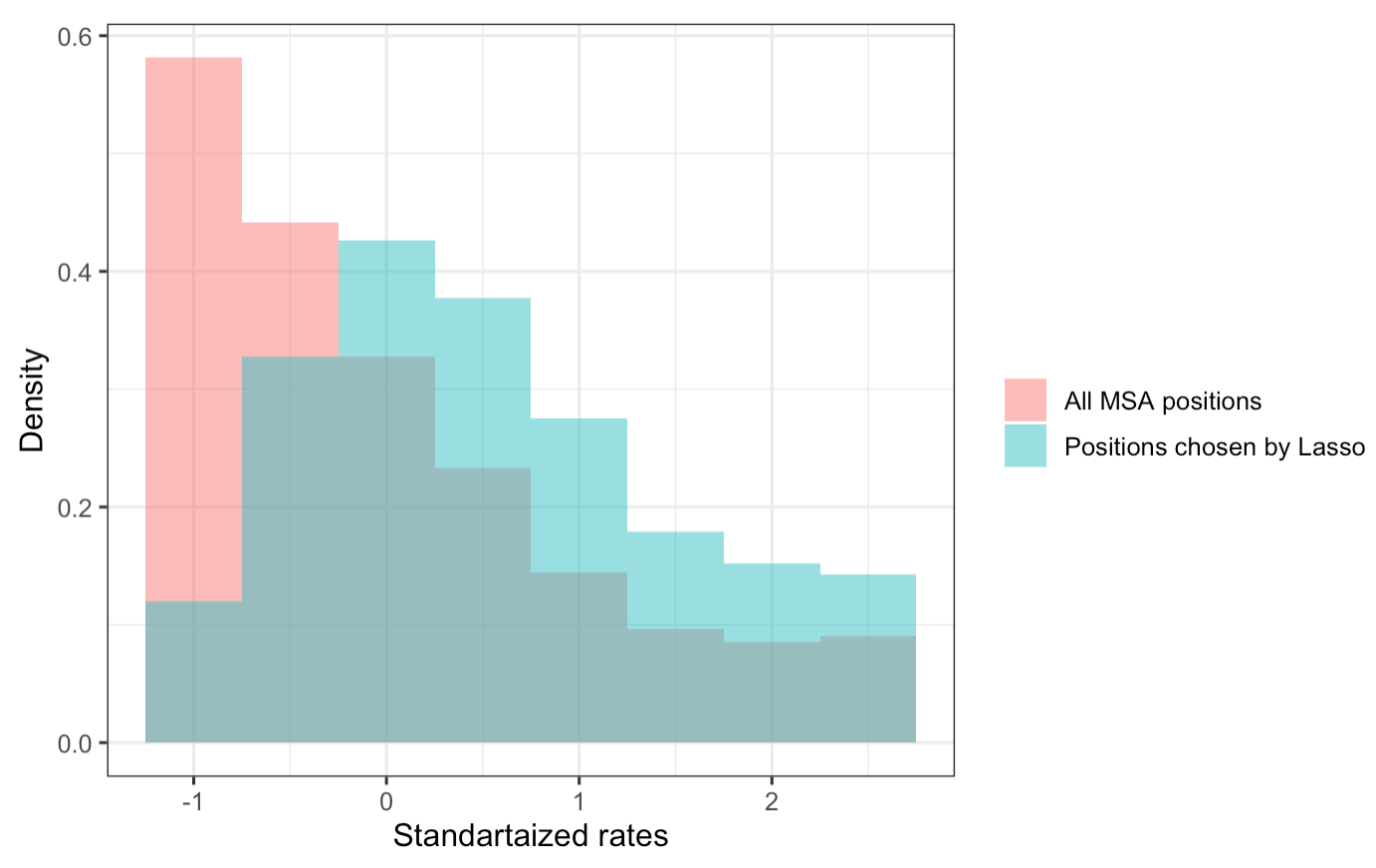
**

**YangA8**

**
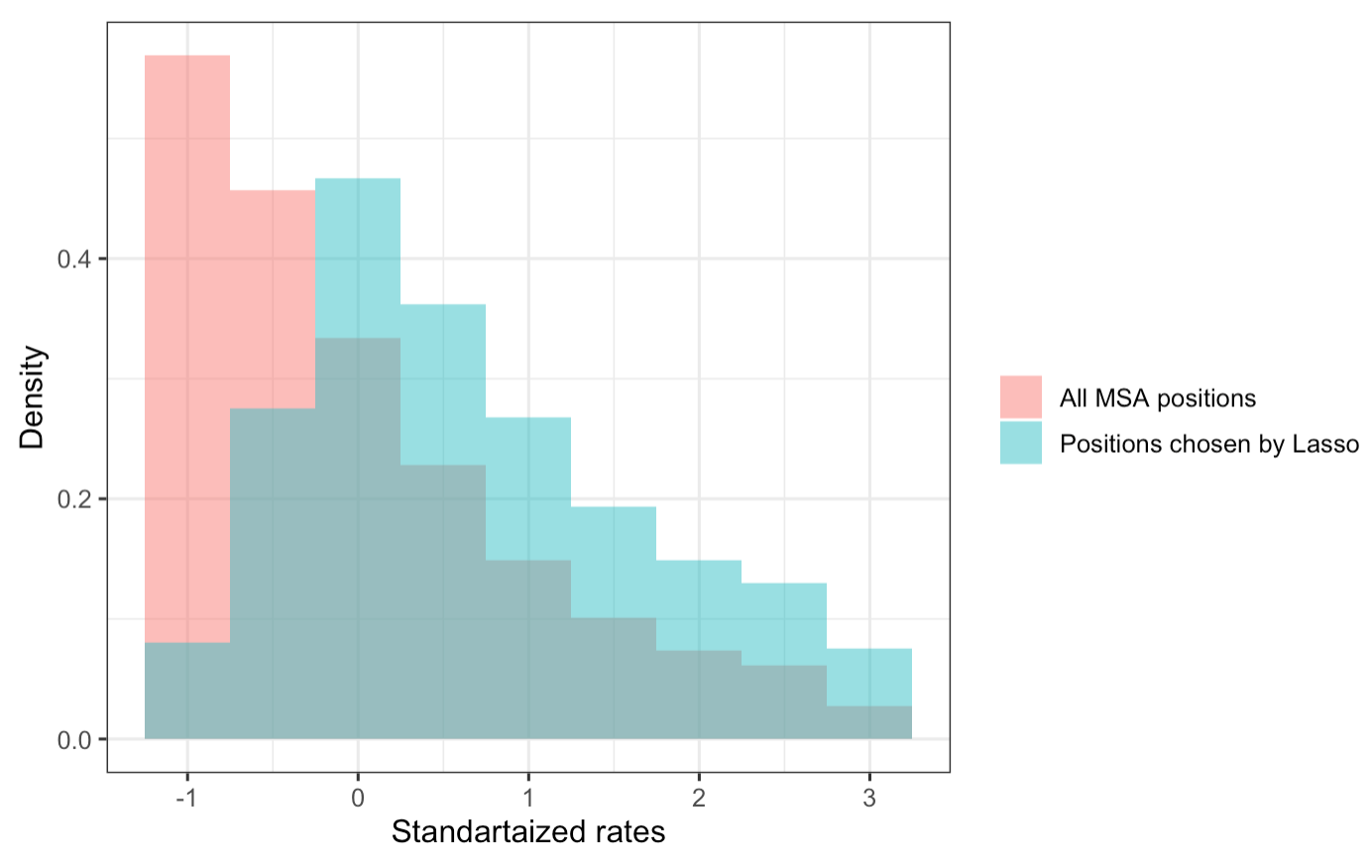
**

**MisoA2**

**
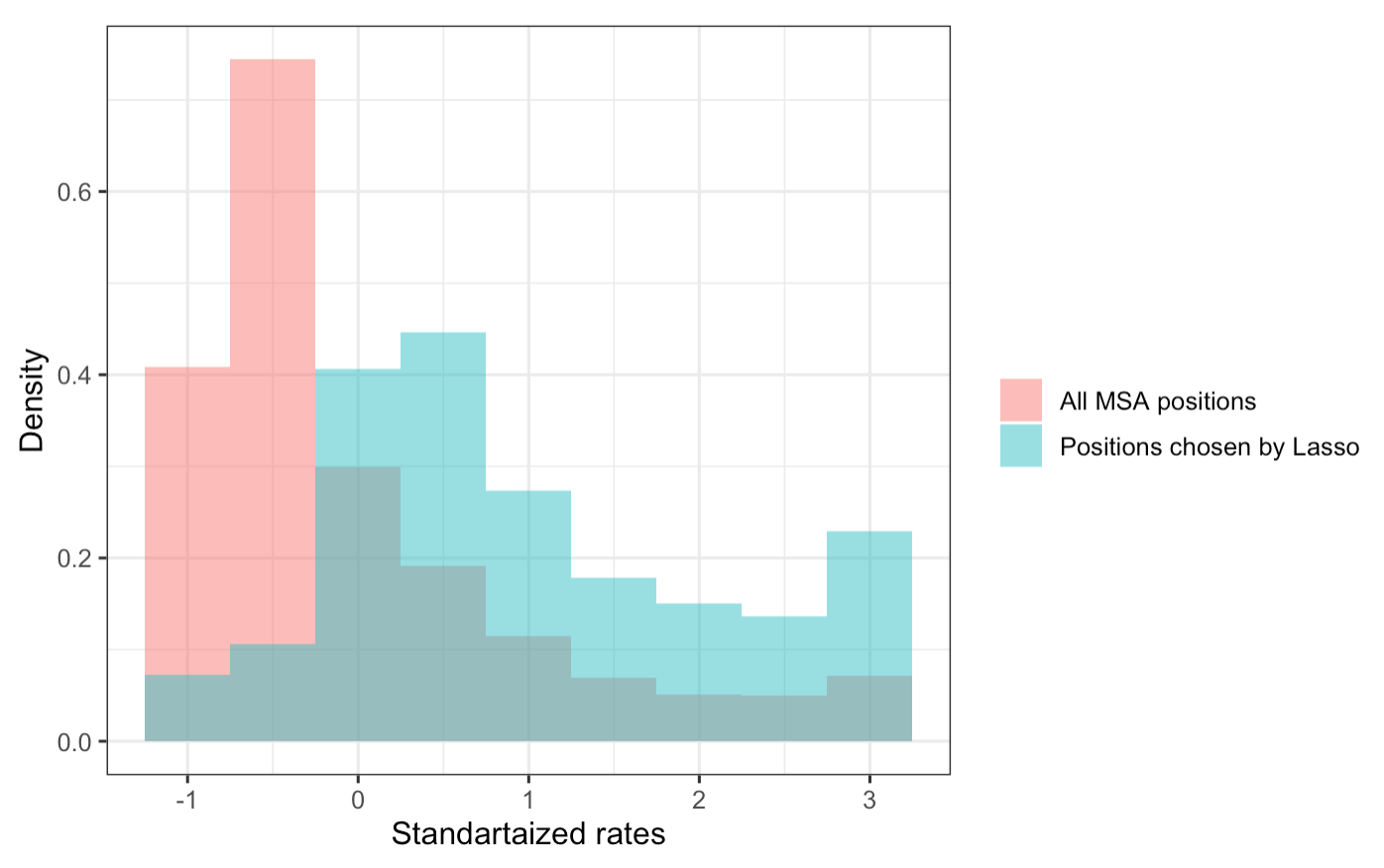
**

**StruA5**

**
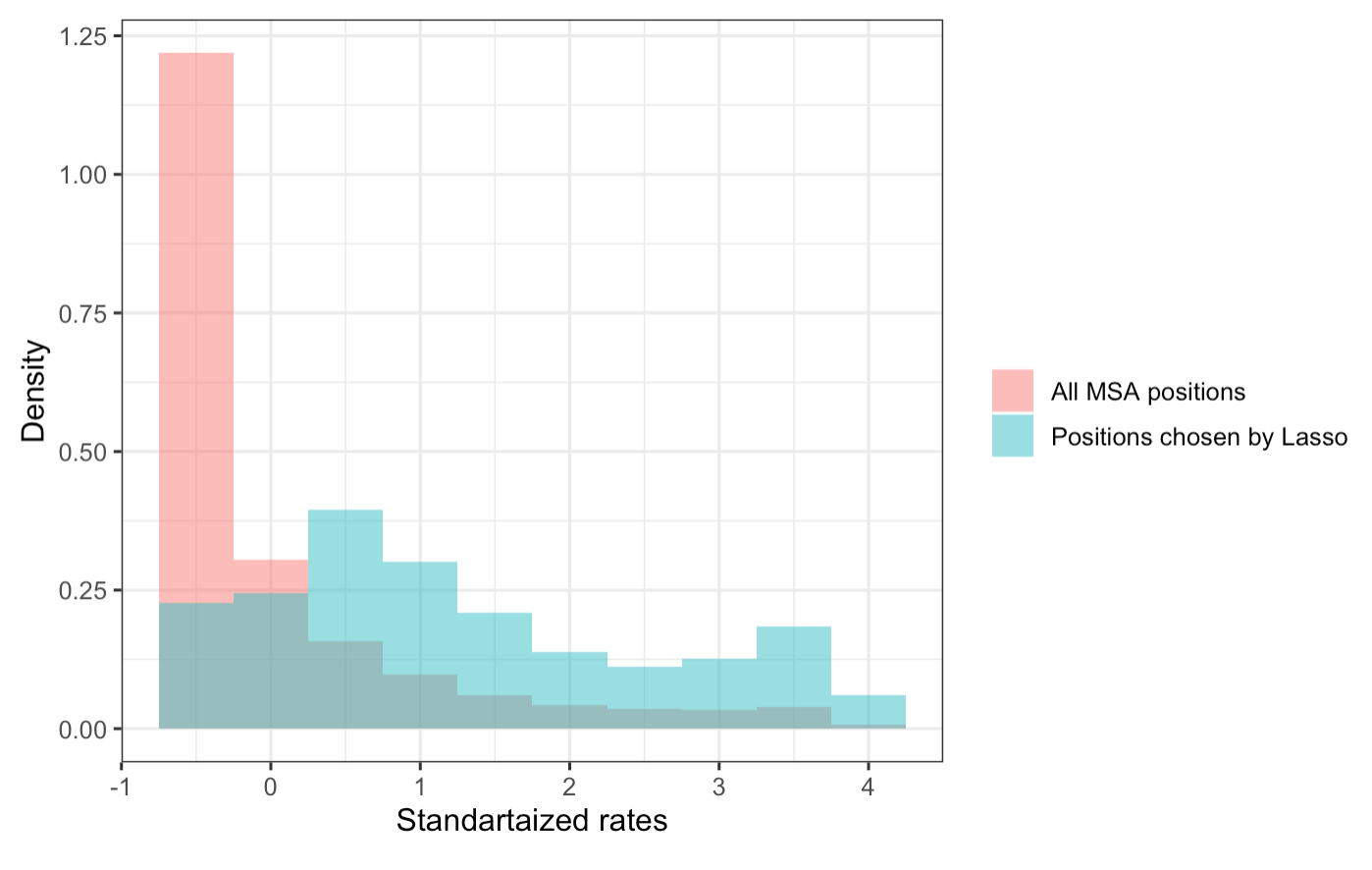
**

**WickA3**

**
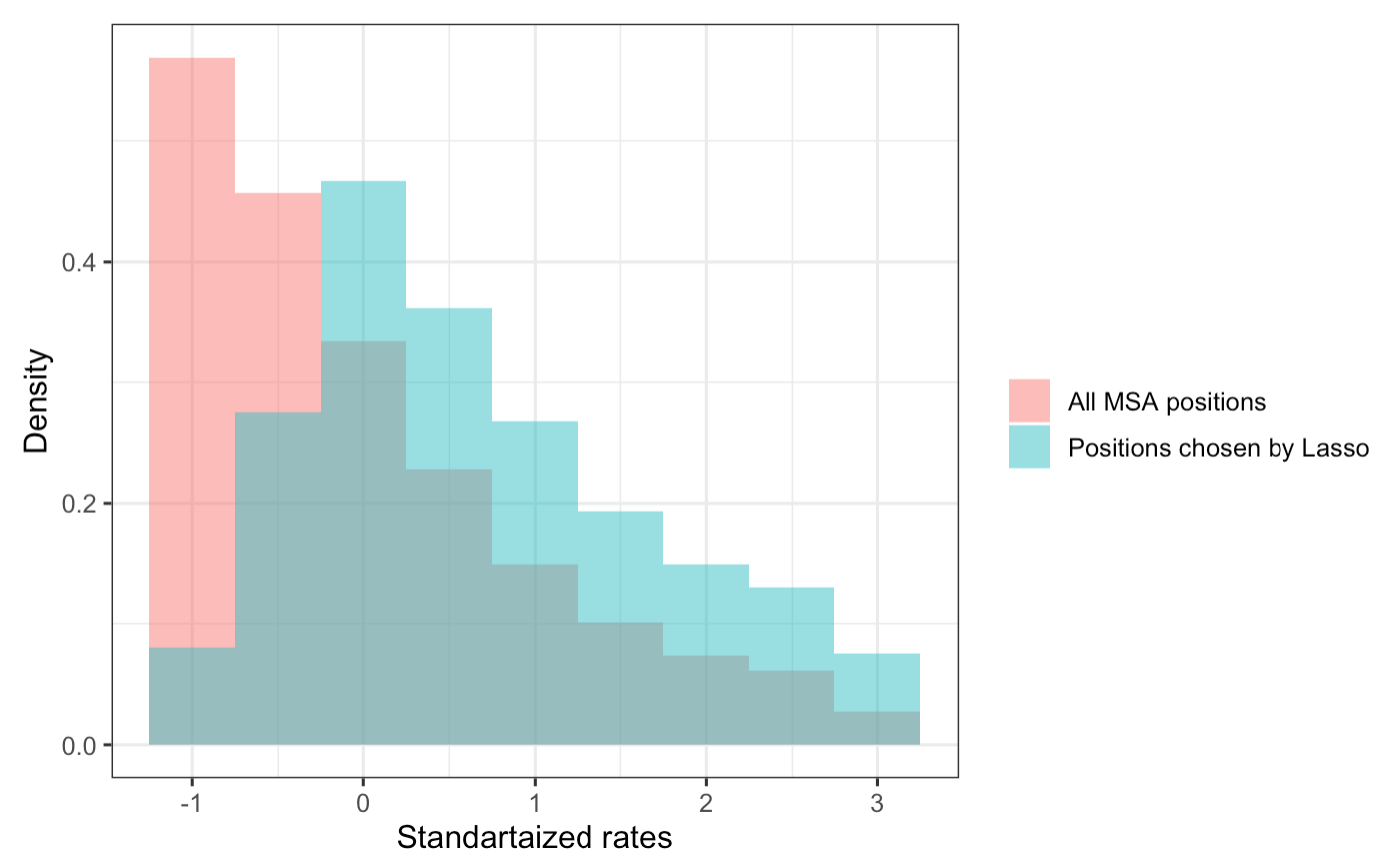
**

# **Figure S2.**

Each of the five panels corresponds to a different alignment. Shown are the distributions of evolutionary rates for the entire alignments against those of the sampled alignments. All datasets have 30 sequences and 80,000 positions and site sampling is performed using ζ = 5% of the positions and $\eta$ *=* 4,000 trees used for training.


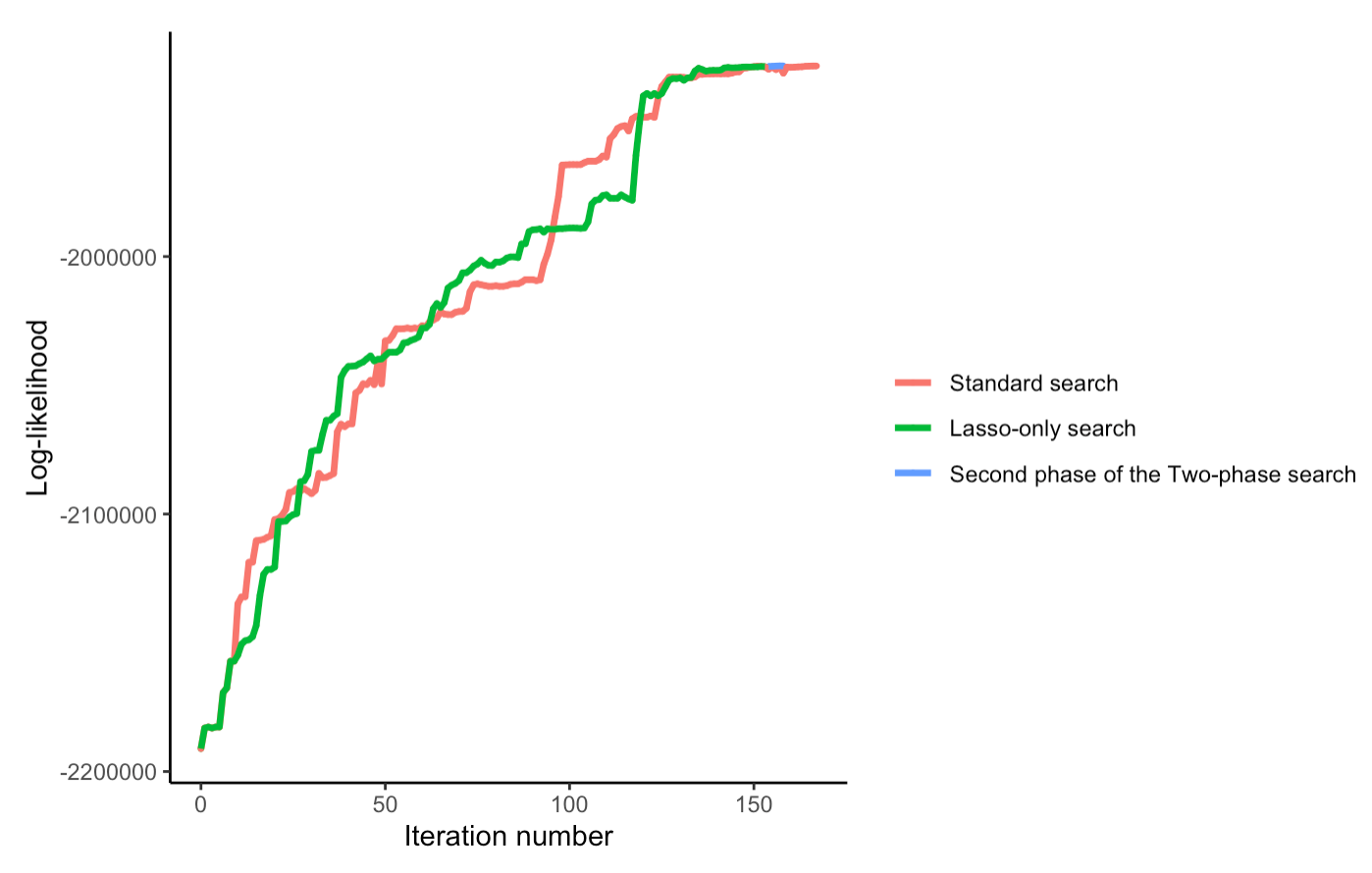


# **Figure S3.**

The log-likelihood at each iteration of the three search strategies on the NagyA1 dataset (30 sequences and 80,000 positions).
